# Supplementary material for: CuentosIE: can a chatbot about “tales with a message” help to teach emotional intelligence?
Source: PeerJ Comput Sci. 2024 Feb 29;10:e1866. doi: 10.7717/peerj-cs.1866 (PMC10909183; doi:10.7717/peerj-cs.1866)
Supplement: Supplemental Information 1 [file peerj-cs-10-1866-s001.pdf]

Opinion sobre CuentosIE

Aquí puedes expresar anónimamente tu experiencia de uso con CuentosIE y tus recomendaciones de mejora

aferrandez3@gmail.com Cambiar de cuenta

No compartido

* Indica que la pregunta es obligatoria

Fecha nacimiento (sólo por motivos estadísticos) *

Fecha

dd/mm/aaaa

Género (sólo por motivos estadísticos) *

- Mujer
- Hombre
- Otro:

Puntuación general a CuentosIE (en su conjunto) *

1	2	3	4	5	6	7	8	9	10
○	○	○	○	○	○	○	○	○	○

Puntuación sobre la opción de **elegir** y buscar cuentos *

1	2	3	4	5	6	7	8	9	10
○	○	○	○	○	○	○	○	○	○

Puntuación sobre la opción de **hablar de emociones** *

1	2	3	4	5	6	7	8	9	10
○	○	○	○	○	○	○	○	○	○

Puntuación sobre **cuánto crees que te ha ayudado a conocer más tus emociones** *

1	2	3	4	5	6	7	8	9	10
○	○	○	○	○	○	○	○	○	○

Opinión general (expresa libremente qué te ha parecido CuentosIE: bien, mal, lo recomendaría, ...)

Tu respuesta

Sugerencias de mejora (cómo mejorarías CuentosIE)

Tu respuesta

Errores que has detectado al utilizar CuentosIE

Tu respuesta

Enviar

Borrar formulario

Nunca envíes contraseñas a través de Formularios de Google.

Este formulario se creó en Universitat d'Alacant / Universidad de Alicante. Notificar uso inadecuado

Google Formularios
