# Supplementary material for: CuentosIE: can a chatbot about “tales with a message” help to teach emotional intelligence?
Source: PeerJ Comput Sci. 2024 Feb 29;10:e1866. doi: 10.7717/peerj-cs.1866 (PMC10909183; doi:10.7717/peerj-cs.1866)
Supplement: Supplemental Information 2 [file peerj-cs-10-1866-s002.pdf]

Opinion about CuentosIE

Here you can anonymously express your experience using CuentosIE and your recommendations for improvement

agrandez3@gmail.com Change account

Not shared

* Indicates that the question is mandatory

Date of birth (for statistical reasons only) *

Date

dd/mm/aaaa

Gender (for statistical reasons only) *

- Women
- Man
- Other:

Overall score for CuentosIE (as a whole) *

1	2	3	4	5	6	7	8	9	10
○	○	○	○	○	○	○	○	○	○

Score on the option to **choose** and search for stories *

1 2 3 4 5 6 7 8 9 10

○ ○ ○ ○ ○ ○ ○ ○ ○ ○

Score on the option to **talk about emotions** *

1 2 3 4 5 6 7 8 9 10

○ ○ ○ ○ ○ ○ ○ ○ ○ ○

Score on **how much you think it has helped you learn more about your emotions** *

1 2 3 4 5 6 7 8 9 10

○ ○ ○ ○ ○ ○ ○ ○ ○ ○

General opinion (freely express what you thought of CuentosIE: good, bad, I would recommend it, ...)

Your answer

Suggestions for improvement (how you would improve CuentosIE)

Your answer

Errors that you have detected when using CuentosIE

Your answer

Send

Delete form

Never send passwords through Google Forms.

This form was created at Universitat d'Alacant / University of Alicante. Report inappropriate use

Google Forms
