# Supplementary material for: CuentosIE: can a chatbot about “tales with a message” help to teach emotional intelligence?
Source: PeerJ Comput Sci. 2024 Feb 29;10:e1866. doi: 10.7717/peerj-cs.1866 (PMC10909183; doi:10.7717/peerj-cs.1866)
Supplement: Supplemental Information 4 [file peerj-cs-10-1866-s004.tgz › index - Antes de presentacion.php]

CuentosIE: chatbot de Cuentos con mensaje para aprender Inteligencia Emocional


*note\_add*


*help\_outline*

### CuentosIE - Usuario no registrado

¡Hola! Con **CuentosIE** podrás leer cuentos con mensaje seleccionados para ayudarte a gestionar y reflexionar sobre las emociones, y mejorar tu **Inteligencia Emocional**.

Podrás **buscar cuentos clasificados por emociones** (calma, tensión, certeza, duda, compasión, ira, diversión, aburrimiento, agrado, malestar, alegría, tristeza, placer, dolor, satisfacción, frustración, deseo, fobia, amor, odio, valentía, miedo, fortaleza, agotamiento, entusiasmo, apatía, arrogancia, humillación), **o por temas psicológicos** (aborto, adicciones, adolescencia, alimentación, anorexia, bulimia, alzheimer, autismo, bullying, acoso, depresión, bipolaridad, educación, covid, envejecimiento, esquizofrenia, estrés, ansiedad, hiperactividad, inmigración, lgtb, muerte, racismo, resiliencia, sexo, suicidio, timidez, trabajo, violencia).

CuentosIE **te ayudará a conocerte mejor** a través de tus emociones y **mejorará tu comprensión lectora**, a través del análisis de los cuentos.

Hay que tener en cuenta que muchas tradiciones espirituales utilizan los cuentos como un medio muy eficaz para contener y transmitir conocimientos y verdades profundas. Además, los cuentos se usan en diferentes ámbitos profesionales: **coaching, innovación empresarial, educación, psicología, …**

Por último, indicarte que CuentosIE se ha diseñado a modo de **red social**, permitiéndote añadir los cuentos que consideres importantes.

CuentosIE: …

Recuerda que **si te registras** (opción  del menú superior) no te pediremos ningún dato personal, ni siquiera tu email. Así **evitaría recomendarte cuentos ya leídos por ti**. Del mismo modo, **seleccionaría cuentos ajustados a tu edad**…

Guardaré nuestras charlas (totalmente anónimas) para mejorar este campo científico. **NO utilizamos cookies con fines publicitarios ni de marketing**.

Puedes **añadir nuevos cuentos a CuentosIE** en la opción del menú superior *note\_add*.

**Envía anonimamente tu opinión**, experiencia de uso y sugerencias de mejora rellenando el siguiente formulario.

**Si en algún momento no consigo entenderte**, por favor pincha en *live\_help* del menú inferior

CuentosIE: …

*live\_help*

#### Modo de contacto con los autores

×

### Envía tu opinión sobre CuentosIE

> Puedes enviar anonimamente tu opinión, experiencia de uso y recomendaciones de mejora rellenando el siguiente formulario.

### Director del proyecto

> Antonio Ferrández Rodríguez .
> Departamento de Lenguajes y Sistemas Informaticos.
> Grupo de Procesamiento del Lenguaje y Sistemas de Información.
> Universidad de Alicante.
> Carretera San Vicente S/N.
> 03080 ALICANTE, España.
> +34-96-590-3400 ext. 2445.

### Equipo científico

- Expertos en procesamiento automático del lenguaje natural: Antonio Ferrández Rodríguez , Jesús Peral Cortés  y Ángel R. Lloret Rivera , profesores del departamento de Lenguajes y Sistemas Informáticos de la Universidad de Alicante
- Doctora en psicología: María del Carmen Rocamora Rodríguez , profesora de la Universidad CEU Cardenal Herrera.

#### Tecnologías usadas en el programa

×

El chatbot CuentosIE es el resultado de diversos proyectos y estudios de investigación realizado por los autores, englobados dentro de los campos de la Inteligencia Artificial (IA) y el Procesamiento del Lenguaje Natural (PLN).

Concretamente en la aplicación del chatbot se utilizan los diferentes analizadores del lenguaje, como son analizadores léxicos, sintácticos y semánticos, incluyendo resolución de anáfora y elipsis. Específicamente usamos FreeLing, así como analizadores desarrollados por los creadores de CuentosIE.

Además, se utilizan aplicaciones propias de Recuperación de Información (buscadores tipo Google) y Clasificadores Automáticos que indexan un conjunto de cuentos disponibles en la Web. Encontrarás el enlace a cada cuento para el caso de querer saber más de los autores de los cuentos.

Estos buscadores se han especializado en el dominio de los cuentos, para alcanzar una mejor precisión, dotándolos de la inteligencia extraída de diferentes fuentes de conocimiento. Por ejemplo, se ha utilizado la clasificación de emociones del artículo "La estructura de la emoción humana: Un modelo cromático del sistema afectivo. Salud Mental, vol. 24, núm. 4, agosto, 2001, pp. 20-35 Instituto Nacional de Psiquiatría Ramón de la Fuente Muñiz Distrito Federal, México". Asimismo, se ha utilizado la clasificación de temas psicológicos de la "American Psychological Association (APA)"

Todas estas herramientas y tecnologías utilizan la información que le ofrece el usuario, a la cual se ajustan mediante técnicas de aprendizaje automático.

#### Ayuda

×

Los Maestros espirituales de la humanidad sabían que, con frecuencia, el ser humano reacciona ante la verdad con recelo y hostilidad, pero que es casi imposible que se resista a un relato. Por eso, desde hace milenios, muchas tradiciones espirituales utilizan los cuentos como un medio muy eficaz para contener y transmitir conocimientos y verdades profundas (link)

Todos los cuentos tienen el potencial de convertirse en un medio terapéutico, las emociones que afloran ofrecen caminos de alivio y esperanza para afrontar las pruebas de la vida. Por eso los cuentos trascienden y llegan al corazón. Cuando escuchamos una historia llega exactamente donde necesitamos y nos permite mediante la metáfora construir nuevas formas de sostener nuestro mundo, cultivando valores positivos, aumentando la resiliencia y la personalidad (link)

---

La popularización del término "inteligencia emocional" se debe a la obra de Daniel Goleman, Inteligencia emocional, publicada en 1995, en el cual se analiza la capacidad de motivarnos a nosotros mismos, de perseverar en el empeño a pesar de las posibles frustraciones, de controlar los impulsos, de diferir las gratificaciones, de regular nuestros propios estados de ánimo, de evitar que la angustia interfiera con nuestras facultades racionales y la capacidad de empatizar y confiar en los demás.

La "educación emocional" es una de las cuestiones pendientes en nuestra sociedad, adivinándose su beneficio potencial en multitud de problemas actuales: bullying, suicidio, violencia de género, estrés, ansiedad, depresión, anorexia, discriminación, autismo, etc.

Con ello, queremos resaltar su importancia tanto para niños, adolescentes o adultos, y para ayudar en esta ardua empresa, se propone este chatbot que establece una interacción con sus usuarios a través de cuentos. El abordarlo a través de los cuentos se justifica por seguir la tradición milenaria del ser humano, la cual se ha demostrado altamente efectiva para transmitir y entender conocimientos, de fácil comprensión gracias a su simplicidad a través de su moraleja o metáforas asociadas.

En cuanto a los grupos beneficiarios y objetivo de este chatbot, se encuadrarían tanto los alumnos, los docentes y profesionales de la salud mental, ya que una de las dificultades principales que encuentran los psicólogos en sus consultas, es el conseguir que los pacientes se abran realmente a la raíz de sus problemas. Gracias al carácter anónimo de internet, se pretende que este chatbot ayude a cualquier tipo de usuario.

---

El chatbot CuentosIE espera que el usuario empiece una conversación, de manera que a partir de lo que éste describa, el chatbot sea capaz de analizar sus emociones, y según ellas, sugerirle cuentos
de acuerdo a una clasificación interna de los cuentos realizada automáticamente.

Otra opción sería indicar explícitamente qué tipo de cuento te interesa leer, mostrándole una serie de sugerencias adecuadas a la edad del usuario.

Ante cada sugerencia CuentosIE le pedirá confirmación de que ese cuento le ha sido útil.

Igualmente, se entablará una conversación sobre el cuento para que mediante una interacción en forma de preguntas el usuario pueda profundizar en el conocimiento extraído del cuento. Así se animará al usuario a expresar sus "problemas o necesidades emocionales" o bien sus intereses sobre temas de "inteligencia emocional".

---

En caso que el usuario se registre en la aplicación, CuentosIE recordará los cuentos que le ha sugerido anteriormente, para evitar su repetición en las sugerencias de cuentos.

#### Configuración de la aplicación

×

###### Cookies

Este programa solo utiliza cookies propias, ninguna cookie de terceros publicitaria o de marketing.

###### Parámetros de configuración


**Número máximo de cuentos a mostrar en cada búsqueda**

**Tamaño del bloque de interacciones con CuentosIE al analizar un cuento**

**Tamaño del bloque de interacciones al hablar con CuentosIE**

Aceptar
Cancelar

#### Añadir nuevos cuentos a CuentosIE

×

Introduce la información del cuento que quieres añadir en CuentosIE

---

**Título del cuento:**

---

**URL del cuento:**

---

**El cuento crees que será de utilidad para:**
  

Todo el mundo

Niños

Adultos

---

**Emociones tratadas en el cuento (pasa el ratón sobre cada emoción en caso de duda):**

Calma

Tensión
  

Certeza

Duda
  

Compasión

Ira
  

Diversión

Aburrimiento
  

Agrado

Malestar
  

Alegría

Tristeza
  

Placer

Dolor
  

Satisfacción

Frustración
  

Deseo

Fobia
  

Amor

Odio
  

Valentía

Miedo
  

Fortaleza

Agotamiento
  

Entusiasmo

Apatía
  

Arrogancia

Humillación

---

**Tema psicológico del que trata el cuento (pasa el ratón sobre cada tipo para mostrarte las definiciones de la RAE):**

aborto

adicciones
  

adolescencia

alimentación (anorexia, bulimia, …)
  

alzheimer

autismo
  

bullying (acoso)

depresión
  

bipolaridad

educación
  

covid

envejecimiento
  

esquizofrenia

estrés/ansiedad
  

hiperactividad

inmigración
  

lgtb

muerte
  

racismo

resiliencia
  

sexo

suicidio
  

timidez

trabajo

violencia

---

**El cuento:**

---

**Moraleja a extraer del cuento:**

---

Aceptar
Cancelar

#### Emociones detectadas

×

###### 

Calma

Tensión
  

Certeza

Duda
  

Compasión

Ira
  

Diversión

Aburrimiento
  

Agrado

Malestar
  

Alegría

Tristeza
  

Placer

Dolor
  

Satisfacción

Frustración
  

Deseo

Fobia
  

Amor

Odio
  

Valentía

Miedo
  

Fortaleza

Agotamiento
  

Entusiasmo

Apatía
  

Arrogancia

Humillación

Aceptar
Cancelar

#### Creación de una nueva cuenta

×

**Nombre de usuario para su identificación (ya que es anónimo, no es necesario el email, valdría cualquier nombre)**

**Password (mínimo longitud 7)** 


**Repetir Password**

**Año de nacimiento (si quieres que te filtremos los cuentos por tu edad)**

**Género**

Hombre

Mujer

Crear nueva cuenta
Cancelar

#### Modificación de una cuenta

×

**Nombre de usuario o email para su identificación**

**Password (mínimo longitud 7)** 


**Repetir Password**

**Año de nacimiento (si quieres que te filtremos los cuentos por tu edad)**

**Género**

Hombre

Mujer

Modificar
Cancelar

#### ¿Quiere borrar esta cuenta?

×

**Nombre de usuario o email para su borrado**

**Año de nacimiento**

**Género**

Hombre

Mujer

Borrar
Cancelar

#### Registro de usuarios

×

Por favor rellene su usuario de identificación (ya que es anónimo, no es necesario el email, valdría cualquier nombre) para así **entrar**, **modificar**, **borrar o bien **crear** una nueva cuenta.**

---

**Nombre de usuario o email**

**Password (mínimo longitud 7)**

---

Entrar
Modificar
Borrar
Crear
Cancelar

#### Ayuda contextual al usuario

×

CuentosIE tiene como objetivo el ayudar a desarrollar la inteligencia emocional a través de cuentos con mensaje, a través de la **búsqueda y el análisis** del cuento más adecuado para el usuario.

Si decides que CuentosIE te **recomiende** un cuento, te animará a que mantengas una conversación para realizar un **análisis de tus emociones**, según las cuales te sugerirá un cuento adecuado para ti. Por otro lado, si decides **elegir** un cuento, podrás afinar la búsqueda del cuento que te apetezca leer (por ejemplo, 'quiero elegir cuentos sobre la ansiedad').

---

**Ahora, puedes hacer:**

Elegir uno de mis cuentos

Recomendarte un cuento especial

---

Aceptar
Cancelar

#### Ayuda contextual al usuario

×

Hola, estamos analizando el cuento

---

**¿Qué prefieres hacer a continuación?:**

Seguir analizando este cuento 
  
Elegir otros cuentos
  
Recomendarte un cuento tras conversar contigo
  
Salir de CuentosIE

---

Aceptar
Cancelar

#### Ayuda contextual al usuario

×

Ahora, te puedo recomendar cuentos según el problema al que estén orientados (por ejemplo: bullying, suicidio, violencia de género, estrés, ansiedad, depresión, anorexia, discriminación, autismo, esquizofrenia, …) o según de qué quieres que trate el cuento (p.ej. sobre animales, el amor, dinero, …)

---

**¿Qué prefieres hacer a continuación?:**

Elegir un cuento según la temática que me digas

Quizás podemos recomendarte un cuento
  
Salir de CuentosIE

---

Aceptar
Cancelar

#### Ayuda contextual al usuario

×

Estamos hablando para que según nuestra conversación pueda recomendarte un cuento especial para ti.

---

**Dime, ¿qué quieres hacer ahora?:**

¿Seguir hablando?
  

¿Te propongo un cuento según nuestra conversación?
  

¿O quieres elegir la temática del cuento?
  

Salir de CuentosIE

---

Aceptar
Cancelar
