# Supplementary material for: CuentosIE: can a chatbot about “tales with a message” help to teach emotional intelligence?
Source: PeerJ Comput Sci. 2024 Feb 29;10:e1866. doi: 10.7717/peerj-cs.1866 (PMC10909183; doi:10.7717/peerj-cs.1866)
Supplement: Supplemental Information 4 [file peerj-cs-10-1866-s004.tgz › index.php]

CuentosIE: chatbot de Cuentos con mensaje para aprender Inteligencia Emocional


*Pruébalo pinchando aquí...*


*Demo de CuentosIE...*

# ***CuentosIE: chatbot de Cuentos con mensaje para aprender Inteligencia Emocional***

Hemos seleccionado y clasificado cuentos especiales por estados emocionales y temas psicológicos

Ponemos a tu disposición un potente buscador para que encuentres el cuento que tú necesitas

Podrás chatear con CuentosIE que te recomendará el más beneficioso para tu estado emocional

Y conocerte mejor a ti mismo, mediante el trabajo y conocimiento de diferentes emociones

Te facilitaremos el conocimiento de diferentes enfermedades mentales mediante cuentos especiales

Podrás profundizar en el conocimiento de temas psicológicos actuales (p.ej. bullying o anorexia)

  
  

La **“educación emocional”** es una de las cuestiones pendientes en nuestra sociedad, adivinándose su beneficio potencial en multitud de problemas actuales: **bullying, suicidio, violencia de género, estrés, ansiedad, depresión, anorexia/bulimia nerviosa, discriminación, autismo,** etc. El abordarla a través de los **cuentos** se justifica por seguir la **tradición milenaria** del ser humano, la cual se ha demostrado altamente efectiva para transmitir y entender conocimientos, de fácil comprensión gracias a su simplicidad a través de su moraleja o metáforas asociadas.

Los **grupos beneficiarios**, objetivo de trabajo de este chatbot, son tanto **alumnos y sus docentes, como cualquier profesional del ámbito de la psicopedagogía y los usuarios de sus servicios**, con la finalidad de dotar a estos profesionales de una herramienta que facilite que los niños y niñas con las que trabajen se relajen, confíen en el profesional y descubran poco a poco el origen de sus problemas con el fin de buscar una solución a los mismos. Gracias al carácter anónimo de internet, se pretende que este chatbot ayude a cualquier tipo de usuario, tanto particular como los que trabajan en una **empresa**, puesto que también es habitual utilizar este tipo de cuentos en sesiones de **coaching** o **gestión de equipos de trabajo** empresariales.

Hay gran variedad de webs dedicadas a la publicación de cuentos, así como trabajos científicos que avalan su utilidad. Este **chatbot** se centra en **recopilar** y **clasificar** los cuentos ***ESPECIALES SELECCIONADOS POR NUESTRO EQUIPO DE PSICÓLOGOS***, **recomendando al usuario** los que le sean útiles, a través de un **analizador de variables emocionales**.

  

**¿Cómo elegir cuentos en CuentosIE?**

**¿Cómo leer y analizar cuentos en CuentosIE?**

**¿Cómo CuentosIE recomienda cuentos a través del análisis de emociones?**

**Añadir nuevos cuentos y registro de usuarios en CuentosIE**

  

## Elegir cuentos en CuentosIE

  

**Al empezar a charlar con el chatbot**, se puede "elegir cuentos sobre ... (p.ej. bullying o estrés)", o "hablar sobre emociones" (para entablar una conversación sobre cómo te encuentras y según el estado emocional que detecte CuentosIE, recomendarte un cuento especial).

Si se **elige** la temática del cuento (por ejemplo, en la imagen se muestra que la búsqueda inicial de "Quiero buscar cuentos sobre enfermedades mentales") se mostrará a continuación una selección de los mismos vinculada con el interés del usuario.

A continuación se puede **afinar la búsqueda**, por ejemplo, indicando "Mejor solo de bipolaridad".

Esto sería un ejemplo, pero CuentosIE permite total **flexibilidad para las búsquedas**. Podrías **buscar por emociones** (*alegría, rabia, tristeza, miedo, asco, sorpresa, orgullo, culpa, vergüenza, …*), **o por temas vinculados a temáticas psicológicas** (*aborto, adicciones, adolescencia, anorexia, bulimia, alzheimer, autismo, bullying, ciberacoso, depresión, bipolaridad, educación, covid, envejecimiento, esquizofrenia, estrés, ansiedad, hiperactividad, inmigración, lgtb, muerte, racismo, resiliencia, sexo, suicidio, …*). Estas búsquedas las puedes repetir cuantas veces quieras, hasta encontrar el cuento más adecuado.

Una vez elegido un cuento, no hay más que pinchar en el enlace mostrado, **para empezar a leerlo** y que CuentosIE nos ayude a analizarlo y comprenderlo.

  

## Leer y analizar cuentos

  

Una vez seleccionado un cuento, se te mostrará en el chatbot, además de permitirte ir a la web de donde se extrajo dicho cuento. A continuación comienza el proceso de **análisis del cuento**, con el objetivo de **facilitar su comprensión y reflexión de sus contenidos**.

CuentosIE **planteará una serie de preguntas**, como las que aparecen en la imagen acerca de la opinión personal sobre el cuento, sobre la **moraleja** del mismo, o sobre las **emociones y temas psicológicos** de los que trata.

Esta batería de preguntas se ha diseñado con el objetivo de **mejorar la comprensión lectora**, así como para **promover el autoconocimiento a través de las emociones**.

CuentosIE facilita el conocimiento de los estados emocionales (p.ej. el odio o el amor) y de temas psicológicos actuales (p.ej. el suicidio o anorexia). **Al pasar el ratón por cada uno de esos términos**, CuentosIE te muestra información útil para entenderlos.

Es importante destacar que la inteligencia de CuentosIE mejorará conforme se vaya usando, así que todavía habrá **situaciones en las que el chatbot no te entienda**. Para solucionar estas situaciones, siempre se deja disponible la opción de pinchar en *live\_help*, tras lo que se redirigirá a las opciones disponibles en ese momento.

Gracias a vuestra interacción con CuentosIE, **se generará un conjunto anónimo de frases y cuentos etiquetados por emociones**, que será enormemente beneficioso para las técnicas de aprendizaje profundo (*Deep Learning*).

  

## Recomendar cuentos a través del análisis de emociones

  

Cuando entramos en la opción de hablar sobre emociones, el chatbot empezará **pidiendo al usuario que cuente cómo está**, pasando a **detectar su estado emocional** desde la conversación mantenida con CuentosIE.

Por ejemplo, en la imagen, el usuario expresa que "ha tenido un día malo en el trabajo", con lo que **CuentosIE detecta emociones de "frustración, decepción o desengaño"**. Además le muestra una **cita interesante para reflexionar**.

La detección del estado emocional del usuario y las citas presentadas irán mejorando conforme más se utilice CuentosIE. Pero en cualquier caso, **está ideado con el objetivo de "animar" al usuario a que se abra, identifique y muestre sus emociones**.

**Esta conversación finalizará** cuando el usuario lo indique en el chatbot (p.ej. "recomiéndame ya el cuento") o pinchando en *live\_help*.

Tras ello, CuentosIE **mostrará las emociones detectadas en la conversación** (modificables por el usuario), y en función de ellas realizará la **selección de cuentos que traten dichas emociones**.

  

## Añadir nuevos cuentos y registro de usuarios

  

CuentosIE funciona a modo de **red social**, por lo que **los usuarios pueden colaborar añadiendo cuentos** que ellos hayan considerado interesantes, indicando para qué emociones creen que podrían ayudar, así como qué temas psicológicos trata dicho cuento. El equipo de CuentosIE los analizará y **los añadirá al buscador de cuentos integrado en el chatbot**, para que de este modo el resto de usuarios puedan acceder al mismo.

Igualmente, se gestiona un **registro de usuarios**, para lo que no se pide ningún dato personal, ni siquiera el email. Así **se evita recomendar cuentos ya leídos**. Del mismo modo, **se seleccionarían cuentos ajustados a la edad del usuario registrado**.

Por último, destacar que se guardan las conversaciones de forma totalmente anónima, con el objetivo de mejorar este campo científico. Igualmente, CuentosIE **NO utiliza cookies de terceros con fines publicitarios ni de marketing**.

  
  

#### Utilización de cookies propias y de terceros por la FECYT

×

Este programa solo utiliza cookies propias, **ninguna cookie de terceros publicitaria o de marketing**, para gestionar el portal y recabar información de análisis, con la finalidad de mejorar nuestros servicios.

Al continuar usando nuestro sitio web, usted acepta dicho uso.

Aceptar
Cancelar
