# Supplementary material for: CuentosIE: can a chatbot about “tales with a message” help to teach emotional intelligence?
Source: PeerJ Comput Sci. 2024 Feb 29;10:e1866. doi: 10.7717/peerj-cs.1866 (PMC10909183; doi:10.7717/peerj-cs.1866)
Supplement: Supplemental Information 4 [file peerj-cs-10-1866-s004.tgz › testBaron.php]

CuentosIE: chatbot de Cuentos con mensaje para aprender Inteligencia Emocional


Tecnologías 

Configuración 
Añadir cuentos*note\_add*


Ayuda *help\_outline*
Contacto 
Usuarios 
Fin *call\_end*

##### *Usuario no registrado*

*live\_help*


#### MAC1. Test Baron

×

Por favor, lee atentamente todas las frases y elige el número de la opción que mejor te describa o que mejor explique cómo te sientes o piensas ante las situaciones que se presentan. Hay cuatro posibles respuestas.

- Si eliges el número 1, significa que **NUNCA** te pasa
- Si eliges el número 2, significa que **A VECES** te pasa
- Si eliges el número 3, significa que **CASI SIEMPRE** te pasa
- Si eliges el número 4, significa que **SIEMPRE** te pasa lo que dice la frase

Ten en cuenta que ninguna respuesta es mejor que otra, por lo que contesta a todas las frases de forma sincera. No existen respuestas "buenas" o "malas", no se trata de una prueba o de un examen.

***LOS RESULTADOS DE ESTA PRUEBA SON CONFIDENCIALES***

---

**Nombre de usuario en CuentosIE:**

---

**P1** Me gusta divertirme.

---

**P2** Entiendo bien cómo se sienten las otras personas.

---

**P3** Puedo estar tranquilo o tranquila cuando me enfado.

---

**P4** Soy feliz.

---

**P5** Me importa lo que le sucede a otras personas.

---

**P6** Me resulta difícil controlar mi ira (furia).

---

**P7** Me resulta fácil decirle a la gente cómo me siento.

---

**P8** Me gusta cada persona que conozco.

---

**P9** Me siento seguro de mí mismo o de mí misma.

---

**P10** Sé cómo se sienten las otras personas.

---

**P11** Sé cómo mantenerme tranquilo o tranquila.

---

**P12** Cuando me hacen preguntas difíciles, trato de responder de distintas formas.

---

**P13** Pienso que la mayoría de las cosas que hago saldrán bien.

---

**P14** Soy capaz de respetar a los demás.

---

**P15** Algunas cosas me enfadan mucho.

---

**P16** Es fácil para mí entender cosas nuevas.

---

**P17** Puedo hablar con facilidad acerca de mis sentimientos.

---

**P18** Tengo buenos pensamientos acerca de todas las personas.

---

**P19** Espero lo mejor.

---

**P20** Tener amigos es importante.

---

**P21** Me peleo con la gente.

---

**P22** Puedo entender preguntas difíciles.

---

**P23** Me gusta sonreír.

---

**P24** Trato de no herir (dañar) los sentimientos de los otros.

---

**P25** Trato de trabajar en un problema hasta que lo resuelvo.

---

**P26** Tengo mal genio.

---

**P27** Nada me incomoda (molesta).

---

**P28** Me resulta difícil hablar de mis sentimientos profundos.

---

**P29** Sé que las cosas saldrán bien.

---

**P30** Ante preguntas difíciles, puedo dar buenas respuestas.

---

**P31** Puedo describir mis sentimientos con facilidad.

---

**P32** Sé cómo pasar un buen momento.

---

**P33** Debo decir la verdad.

---

**P34** Cuando quiero puedo encontrar muchas formas de contestar a una pregunta difícil.

---

**P35** Me enojo o enfado con facilidad.

---

**P36** Me gusta hacer cosas para los demás.

---

**P37** No soy muy feliz.

---

**P38** Puedo resolver problemas de diferentes maneras.

---

**P39** Tienen que pasarme muchas cosas para que me enfade.

---

**P40** Me siento bien conmigo mismo.

---

**P41** Hago amigos con facilidad.

---

**P42** Pienso que soy el mejor o la mejor en todo lo que hago.

---

**P43** Es fácil para mí decirle a la gente lo que siento.

---

**P44** Cuando contesto preguntas difíciles, trato de pensar en muchas soluciones.

---

**P45** Me siento mal cuando se hieren (dañan} los sentimientos de otras personas.

---

**P46** Cuando me enfado con alguien, me enfado durante mucho tiempo.

---

**P47** Soy feliz con el tipo de persona que soy.

---

**P48** Se me da bien resolver problemas.

---

**P49** Me resulta difícil esperar mi turno.

---

**P50** Me entretienen las cosas que hago.

---

**P51** Me gustan mis amigos.

---

**P52** No tengo días malos.

---

**P53** Tengo problemas para hablar de mis sentimientos a los demás.

---

**P54** Me molesto con facilidad.

---

**P55** Puedo darme cuenta cuando uno de mis mejores amigos no es feliz.

---

**P56** Me gusta mi cuerpo.

---

**P57** Aun cuando las cosas se ponen difíciles, no me doy por vencido o vencida.

---

**P58** Cuando me enfado, actúo sin pensar.

---

**P59** Sé cuando la gente está enfadada, incluso cuando no dicen nada.

---

**P60** Me gusta cómo me veo.


---

Aceptar
Cancelar
