# Supplementary material for: CuentosIE: can a chatbot about “tales with a message” help to teach emotional intelligence?
Source: PeerJ Comput Sci. 2024 Feb 29;10:e1866. doi: 10.7717/peerj-cs.1866 (PMC10909183; doi:10.7717/peerj-cs.1866)
Supplement: Supplemental Information 4 [file peerj-cs-10-1866-s004.tgz › testMayerSalovey.php]

CuentosIE: chatbot de Cuentos con mensaje para aprender Inteligencia Emocional


Tecnologías 

Configuración 
Añadir cuentos*note\_add*


Ayuda *help\_outline*
Contacto 
Usuarios 
Fin *call\_end*

##### *Usuario no registrado*

*live\_help*


#### MAC1. Test Mayer-Salovey

×

Por favor, lee atentamente todas las preguntas

Ten en cuenta que ninguna respuesta es mejor que otra, por lo que contesta a todas las frases de forma sincera. No existen respuestas "buenas" o "malas", no se trata de una prueba o de un examen.

***LOS RESULTADOS DE ESTA PRUEBA SON CONFIDENCIALES***

---

**Nombre de usuario en CuentosIE:**

---

Sección A

---

**A\_1**


Observe la cara de la imagen e indique en qué grado expresa los sentimientos que aparecen a continuacion. (Por favor, seleccione una opción para cada sentimiento y márquela)

Ausencia de felicidad (1) a felicidad extrema (5):

Ausencia de miedo (1) a miedo extremo (5):

Ausencia de sorpresa (1) a sorpresa extremo (5):

Ausencia de asco (1) a asco extremo (5):

Ausencia de entusiasmo (1) a entusiasmo extremo (5):

---

**A\_2**


Observe la cara de la imagen e indique en qué grado expresa los sentimientos que aparecen a continuacion. (Por favor, seleccione una opción para cada sentimiento y márquela)

Ausencia de felicidad (1) a felicidad extrema (5):

Ausencia de tristeza (1) a tristeza extremo (5):

Ausencia de miedo (1) a miedo extremo (5):

Ausencia de sorpresa (1) a sorpresa extremo (5):

Ausencia de entusiasmo (1) a entusiasmo extremo (5):

---

**A\_3**


Observe la cara de la imagen e indique en qué grado expresa los sentimientos que aparecen a continuacion. (Por favor, seleccione una opción para cada sentimiento y márquela)

Ausencia de felicidad (1) a felicidad extrema (5):

Ausencia de tristeza (1) a tristeza extremo (5):

Ausencia de miedo (1) a miedo extremo (5):

Ausencia de sorpresa (1) a sorpresa extremo (5):

Ausencia de entusiasmo (1) a entusiasmo extremo (5):

---

**A\_4**


Observe la cara de la imagen e indique en qué grado expresa los sentimientos que aparecen a continuacion. (Por favor, seleccione una opción para cada sentimiento y márquela)

Ausencia de felicidad (1) a felicidad extrema (5):

Ausencia de tristeza (1) a tristeza extremo (5):

Ausencia de miedo (1) a miedo extremo (5):

Ausencia de enojo (1) a enojo extremo (5):

Ausencia de asco (1) a asco extremo (5):

---

Sección B

Por favor, seleccione el grado de utilidad de cada una de las emociones en las situaciones que aparecen a continuación de acuerdo a la siguiente escala: **Nada útil (1) Muy útil (5)**

---

**B\_1**

¿Qué estado de ánimo sería útil sentir cuando preparamos unos adornos nuevos y emotivos para una fiesta de cumpleaños?:

Fastidio

Aburrimiento

Alegría

---

**B\_2**

¿Qué estado de ánimo seria útil sentir cuando componemos una marcha militar animada?:

Enojo

Entusiasmo

Frustración

---

**B\_3**

¿Qué estado de ánimo sería útil sentir cuando preparamos una receta de cocina complicada y difícil?:

Tensión

Pena

Estado de ánimo neutral

---

**B\_4**

¿Qué estado de ánimo sería útil sentir cuando tratamos de entender qué causó una pelea entre tres niños? Cada niño cuenta una historia diferente sobre cómo empezó la pelea. Averiguar qué ocurrió requiere atender a los detalles de las historias y evaluar los hechos:

Felicidad

Sorpresa

Tristeza

---

**B\_5**

¿Qué estado de ánimo seria útil que sintiera un médico cuando selecciona un tratamiento para un paciente con un tumor cancerigeno? El doctor debe aplicar varios procedimientos eficaces, pero conflictivos entre si al tratar el tumor:

Felicidad

Estado de ánimo neutral

Enojo y desafío

---

Sección C

Seleccione la que, según su opinión, sea la mejor alternativa en cada uno de los siguientes enunciados y márquela.

---

**C\_1**

Marisa se sentía cada vez más y más avergonzada, y comenzó a sentirse despreciable. Más tarde, se sintió: **1. abrumada; 2. deprimida; 3. avergonzada; 4. cohibida; 5. inquieta**

---

**C\_2**

Francisco se sentía contento cuando pensaba en su vida y cuanto más reflexionaba sobre las cosas buenas que había hecho y la alegría que había provocado en los demás, más se sentía: **1. sorprendido; 2. deprimido; 3. aceptado; 4. feliz; 5. asombrado**

---

**C\_3**

Natalia nunca había estado tan sorprendida en su vida. Pero cuando se recuperó un poco de la conmoción por la pérdida y se dio cuenta de que podía beneficiarse de la situación si reflexionaba cuidadosamente, ella: **1. se sintió asombrada; 2. se sintió confusa; 3. negó la situación; 4. se sintió expectante; 5. se sintió pensativa**

---

**C\_4**

Nacho estaba triste por las noticias que había recibido de su familia y quería expresar su dolor más profundo. Cuando supo que no se lo habían contado de inmediato y que el problema era peor de lo que pensaba en un principio, sintió: **1. enojo y sorpresa; 2. tristeza y expectación; 3. conmoción y pena; 4. miedo y aversión; 5. enojo y pesar**

---

**C\_5**

Rafa suele estar bastante contento en el trabajo y las cosas también le van bien en casa. Pensaba que tanto él como sus compañeros de trabajo recibían un buen sueldo y un trato justo. Hoy todo su equipo, incluido él, recibió un aumento de sueldo como parte de los ajustes salariales de la empresa. Rafa se sintió: **1. sorprendido y conmocionado; 2. tranquilo y calmado; 3. satisfecho y eufórico; 4. humillado y culpable; 5. orgulloso y dominante**

---

**C\_6**

Gloria amaba a Javier, sentía que solo le pertenecía a ella. Empezó a verlo como alguien ideal para ella y que rozaba la perfección. Ella: **1. lo respetaba; 2. lo admiraba; 3. lo envidiaba; 4. lo adoraba; 5. le tenía rencor**

---

**C\_7**

Tamara estaba disgustada porque un compañero de trabajo se atribuyó el mérito de un proyecto, y cuando volvió a hacerlo, se sintió: **1. disgustada; 2. enfadada; 3. frustrada; 4. sobresaltada; 5. deprimida**

---

**C\_8**

Después de que le robaran el coche una vez, Carlos instaló una alarma en su nuevo coche. Cuando se lo robaron otra vez, primero se sintió conmocionado y sorprendido y luego sintió: **1. asombro y estupefacción; 2. impotencia, desesperación y enojo; 3. enojo e indignación; 4. celos y envidia; 5. depresión y desprecio**

---

**C\_9**

Cuando Esteban descubrió que varios estudiantes estaban copiándose en los exámenes, pensó que eso estaba moralmente mal. Cuando se lo dijo al profesor, le contestó que él no podia hacer nada. Esteban pensó en llevar el problema ante el jefe de estudios del colegio porque se sentía por lo que había ocurrido: **1. inquieto; 2. enfurecido; 3. indignado; 4. deprimido; 5. entristecido**

---

**C\_10**

Marcos estaba disgustado porque un buen amigo había herido sus sentimientos. Marcos le dijo a su amigo cómo se había sentido y cuando su amigo le ofendió de nuevo, Marcos se sintió: **1. disgustado; 2. temeroso; 3. muy enfadado; 4. preocupado; 5. enfurecido**

---

**C\_11**

Teresa estaba viendo la televisión para seguir la evolución de la gran tormenta que se estaba acercando a la costa en la que vivían sus padres. Conforme la tormenta se dirigía hacia la casa de sus padres se iba sintiendo ansiosa e impotente. En el último minuto, sin embargo, la tormenta cambió de dirección, quedando a salvo toda esa zona del litoral. Ella sintió: **1. alivio y gratitud; 2. sorpresa y conmoción; 3. tensión y alivio; 4. expectación y ansiedad; 5. expectación y calma**

---

**C\_12**

Una mujer que se sentía segura de sí misma y aceptada por los demás, más tarde se sintió deprimida. ¿Qué ocurrió entre el primer sentimiento y el segundo?: **1. Recibió un cumplido dirigido a otra persona; 2. Descubrió que su marido la estaba engañando; 3. Un amigo se puso enfermo; 4. Un paquete que había enviado a un amigo fue entregado a otra persona; 5. Estaba frustrada por el trabajo tan malo que había realizado en un proyecto**

---

**C\_13**

Un niño que esperaba alegremente que llegara su fiesta de cumpleaños se sintió triste una vez transcurrida ésta. ¿Qué pudo ocurrirle para que cambiara de esa manera?: **1. Un gamberro le insultó y él se defendió; 2. Dos amigos que esperaba que vinieran a la fiesta no lo hicieron; 3. Comió demasiado pastel; 4. Su madre le avergonzó delante de otros niños; 5. Su padre lo acusó de algo que no había hecho**

---

**C\_14**

Una mujer de mediana edad se encontraba feliz y poco después se sintió rechazada. ¿Qué pudo ocurrirle para que cambiara de esa manera?: **1. Su hijo tuvo un pequeño accidente en el trabajo; 2. Se dio cuenta que había herido los sentimientos de un buen amigo; 3. Su nuera llegó tarde a la cena Familiar; 4. Su marido la criticó; 5. Perdió un libro importante para ella**

---

**C\_15**

Un hombre se encontraba relajado y posteriormente sintió admiración. ¿Qué pudo ocurrirle para que cambiara de esa manera?: **1. Mientras estaba relajado, resolvió un problema laboral importante; 2. Escuchó una historia sobre un deportista que había establecido un nuevo récord mundial; 3. Su amigo llamó para decirle que acababa de comprarse un coche deportivo a un buen precio; 4. Llegó un paquete con un regalo de su madre; 5. Su médico llamo para decirle que su revisión indicaba que gozaba de buena salud**

---

**C\_16**

Los sentimientos de una mujer se transformaron de expectación en amor. ¿Qué pudo provocar ese cambio?: **1. Realizó una donación y pensó en toda la gente a la que ayudaría; 2. Se compró un vestido que le favorecía mucho; 3. Leyó una revista sobre una estrella de cine que le resultaba muy atractiva; 4. Su madre la llamó para decirle que le iba a mandar un regalo de cumpleaños sorprendente; 5. Fue a una cita y descubrió que tenía muchas cosas en común con un hombre atractivo**

---

**C\_17**

Un ejecutivo de una compañia estaba disgustado y más tarde se sintió ofendido. ¿Qué pudo ocurrirle?: **1. Un subordinado no consiguió los objetivos de ventas establecidos para ese periodo; 2. Otro ejecutivo de la compañía, al que consideraba un incompetente, obtuvo un aumento de sueldo mayor; 3. Leyó un artículo sobre las personas que, en otra parte del mundo, viven en la pobreza y cómo una importante ONG se estaba enfrentando a muchos obstáculos en sus esfuerzos por ayudarles; 4. Su mujer estaba ayudando a sus hijos con los deberes; 5. Parecía que no caía bien a nadie**

---

**C\_18**

Una mujer estaba enojada y seguidamente se sintió culpable. ¿Qué pudo ocurrirle?: **1. Perdió el número de teléfono de un buen amigo; 2. No terminó un trabajo tan bien como esperaba, porque no había tenido el tiempo suficiente; 3. Expresó su enojo a una amiga y luego descubrió que su amiga no había hecho nada para herirla; 4. Perdió a una buena amiga; 5. Estaba enojada porque alguien cotilleaba sobre ella y luego descubrió que todos los demás hacían lo mismo**

---

**C\_19**

Luis apreciaba a su amigo Pedro y poco después lo despreciaba. ¿Qué pudo ocurrirle?: **1. Pedro le perdió un libro muy caro que le había prestado; 2. Pedro fue infiel a su propia mujer; 3. Pedro consiguió un aumento de sueldo que no merecia; 4. Pedro le dijo que se mudaba de ciudad; 5. Luis sintió que había herido a Pedro aunque, en parte, la culpa era de este último**

---

**C\_20**

Una mujer amaba a una persona y luego se sintió segura. ¿Qué pudo ocurrirle?: **1. Se dio cuenta de que la otra persona también la amaba; 2. Decidió no expresar sus sentimientos; 3. Su amor se desvaneció; 4. Le dijo a la otra persona que la amaba; 5. El amor que sentía le dio seguridad**

---

Sección D

Seleccione una opción en cada una de las acciones y márquela.

---

**D\_1**

María se levantó sintiéndose bastante bien. Había dormido estupendamente, se encontraba muy descansada y no tenía ningún tipo de inquietud o preocupación. ¿Cómo ayudaría cada una de estas acciones a mantener su estado de ánimo?:

Acción 1: Se levantó y disfrutó del resto del día (**1. Muy ineficaz 2. Algo ineficaz 3. Ni eficaz, ni ineficaz 4. Algo eficaz 5. Muy eficaz**)

Acción 2: María disfrutó de ese sentimiento y decidió pensar y apreciar todas las cosas que le iban bien (**1. Muy ineficaz 2. Algo ineficaz 3. Ni eficaz, ni ineficaz 4. Algo eficaz 5. Muy eficaz**)

Acción 3: Decidió que era mejor ignorar el sentimiento porque de todos modos no duraría mucho (**1. Muy ineficaz 2. Algo ineficaz 3. Ni eficaz, ni ineficaz 4. Algo eficaz 5. Muy eficaz**)

Acción 4: Aprovechó ese sentimiento positivo para llamar a su madre, que había estado deprimida, e intentó animarla (**1. Muy ineficaz 2. Algo ineficaz 3. Ni eficaz, ni ineficaz 4. Algo eficaz 5. Muy eficaz**)

---

**D\_2**

Andrés trabaja tan duro, incluso más, que uno de sus colegas. De hecho, por lo general sus ideas son mejores y obtiene resultados muy positivos para la compañía. Su colega hace un trabajo mediocre pero se implica en la política de la empresa de tal manera que progresa. Así, cuando el jefe de Andrés anunció que el premio al mérito anual sería para su colega, Andrés se enojó mucho. En qué grado serían eficaces cada una de estas acciones para ayudar a Andrés a sentirse mejor?:

Acción 1: Andrés se tomó un descanso y recapacitó sobre las cosas buenas de su vida y su trabajo (**1. Muy ineficaz 2. Algo ineficaz 3. Ni eficaz, ni ineficaz 4. Algo eficaz 5. Muy eficaz**)

Acción 2: Hizo una lista de las características positivas y negativas de su colega (**1. Muy ineficaz 2. Algo ineficaz 3. Ni eficaz, ni ineficaz 4. Algo eficaz 5. Muy eficaz**)

Acción 3: Se sintió fatal por comportarse de esa manera y se dijo que no conseguiría nada enfadándose por algo que escapaba a su control (**1. Muy ineficaz 2. Algo ineficaz 3. Ni eficaz, ni ineficaz 4. Algo eficaz 5. Muy eficaz**)

Acción 4: Andrés decidió decirle a la gente que su colega había realizado un trabajo muy pobre y que, por tanto, no se merecía el premio. Reunió documentos e informes para demostrar su punto de vista y así fundamentar su palabra (**1. Muy ineficaz 2. Algo ineficaz 3. Ni eficaz, ni ineficaz 4. Algo eficaz 5. Muy eficaz**)

---

**D\_3**

Juana no sabía cuándo vencían sus facturas, cuántas más le llegarían en breve ni si podría pagarlas. Además, su coche empezó a hacer unos ruidos extraños y el mecánico le dijo que le iba a costar tanto arreglarlo que no merecía la pena. Ahora no puede quedarse dormida con facilidad, se despierta varias veces durante la noche y está preocupada todo el tiempo. ¿En qué grado serían eficaces cada una de las siguientes acciones para reducir su preocupación?:

Acción 1: Juana intentó calcular cuánto debía y cuándo pagarlo (**1. Muy ineficaz 2. Algo ineficaz 3. Ni eficaz, ni ineficaz 4. Algo eficaz 5. Muy eficaz**)

Acción 2: Juana aprendió técnicas de relajación profunda para calmarse (**1. Muy ineficaz 2. Algo ineficaz 3. Ni eficaz, ni ineficaz 4. Algo eficaz 5. Muy eficaz**)

Acción 3: Juana consiguió el nombre de un contable para que le ayudase a aprender cómo manejar sus finanzas (**1. Muy ineficaz 2. Algo ineficaz 3. Ni eficaz, ni ineficaz 4. Algo eficaz 5. Muy eficaz**)

Acción 4: Decidió buscar un trabajo en el que le pagaran más dinero (**1. Muy ineficaz 2. Algo ineficaz 3. Ni eficaz, ni ineficaz 4. Algo eficaz 5. Muy eficaz**)

---

**D\_4**

A Eduardo nada le va bien. No hay muchas cosas en su vida que le diviertan o le hagan disfrutar. Durante el próximo año, ¿en qué grado serían eficaces cada una de las siguientes acciones para hacer que Eduardo se sintiese mejor?

Acción 1: Eduardo llamó a unos amigos con los que no hablaba desde hacía tiempo e hizo planes para visitarlos (**1. Muy ineficaz 2. Algo ineficaz 3. Ni eficaz, ni ineficaz 4. Algo eficaz 5. Muy eficaz**)

Acción 2: Empezó a comer mejor, a acostarse más temprano y a hacer más ejercicio (**1. Muy ineficaz 2. Algo ineficaz 3. Ni eficaz, ni ineficaz 4. Algo eficaz 5. Muy eficaz**)

Acción 3: Eduardo notaba que deprimía a la gente y decidió estar solo hasta que entendiera lo que le estaba sucediendo. Sentía que necesitaba tiempo para estar consigo mismo (**1. Muy ineficaz 2. Algo ineficaz 3. Ni eficaz, ni ineficaz 4. Algo eficaz 5. Muy eficaz**)

Acción 4: Se dio cuenta de que relajarse delante de la TV por la noche, con una o dos cervezas, le ayudaba a sentirse mejor (**1. Muy ineficaz 2. Algo ineficaz 3. Ni eficaz, ni ineficaz 4. Algo eficaz 5. Muy eficaz**)

---

**D\_5**

Mientras Roberto conducía a casa desde el trabajo se le cruzó un camión enorme. No tuvo tiempo ni para tocar el claxon. Roberto rápidamente giró a la derecha para evitar el choque. Estaba furioso. ¿En qué grado serían eficaces cada una de las siguientes acciones para manejar su enojo?

Acción 1: Roberto se vengó del camionero atravesándose en su camino unos cuantos kilómetros después (**1. Muy ineficaz 2. Algo ineficaz 3. Ni eficaz, ni ineficaz 4. Algo eficaz 5. Muy eficaz**)

Acción 2: Roberto aceptó que esas cosas ocurren y condujo hacia su casa (**1. Muy ineficaz 2. Algo ineficaz 3. Ni eficaz, ni ineficaz 4. Algo eficaz 5. Muy eficaz**)

Acción 3: Gritó tanto como pudo, maldiciendo e insultando al camionero (**1. Muy ineficaz 2. Algo ineficaz 3. Ni eficaz, ni ineficaz 4. Algo eficaz 5. Muy eficaz**)

Acción 4: Juró que nunca volvería a conducir por esa autovia (**1. Muy ineficaz 2. Algo ineficaz 3. Ni eficaz, ni ineficaz 4. Algo eficaz 5. Muy eficaz**)

---

Sección E

---

**E\_1**


Observe la imagen e indique en qué grado expresa los sentimientos que aparecen a continuación. (Por favor, seleccione una opción para cada emoción y márquela en la hoja de respuestas)

Ausencia de felicidad (1) a felicidad extrema (5):

Ausencia de tristeza (1) a tristeza extremo (5):

Ausencia de miedo (1) a miedo extremo (5):

Ausencia de enojo (1) a enojo extremo (5):

Ausencia de asco (1) a asco extremo (5):

---

**E\_2**


Observe la imagen e indique en qué grado expresa los sentimientos que aparecen a continuación. (Por favor, seleccione una opción para cada emoción y márquela en la hoja de respuestas)

Ausencia de tristeza (1) a tristeza extrema (5):

Ausencia de enojo (1) a enojo extremo (5):

Ausencia de sorpresa (1) a sorpresa extremo (5):

Ausencia de asco (1) a asco extremo (5):

Ausencia de entusiasmo (1) a entusiasmo extremo (5):

---

**E\_3**


Observe la imagen e indique en qué grado expresa los sentimientos que aparecen a continuación. (Por favor, seleccione una opción para cada emoción y márquela en la hoja de respuestas)

Ausencia de felicidad (1) a felicidad extrema (5):

Ausencia de miedo (1) a miedo extremo (5):

Ausencia de enojo (1) a enojo extremo (5):

Ausencia de sorpresa (1) a sorpresa extremo (5):

Ausencia de asco (1) a asco extremo (5):

---

**E\_4**


Observe la imagen e indique en qué grado expresa los sentimientos que aparecen a continuación. (Por favor, seleccione una opción para cada emoción y márquela en la hoja de respuestas)

Ausencia de tristeza (1) a tristeza extrema (5):

Ausencia de miedo (1) a miedo extremo (5):

Ausencia de enojo (1) a enojo extremo (5):

Ausencia de sorpresa (1) a sorpresa extremo (5):

Ausencia de asco (1) a asco extremo (5):

---

**E\_5**


Observe la imagen e indique en qué grado expresa los sentimientos que aparecen a continuación. (Por favor, seleccione una opción para cada emoción y márquela en la hoja de respuestas)

Ausencia de felicidad (1) a felicidad extrema (5):

Ausencia de tristeza (1) a tristeza extremo (5):

Ausencia de miedo (1) a miedo extremo (5):

Ausencia de enojo (1) a enojo extremo (5):

Ausencia de asco (1) a asco extremo (5):

---

**E\_6**


Observe la imagen e indique en qué grado expresa los sentimientos que aparecen a continuación. (Por favor, seleccione una opción para cada emoción y márquela en la hoja de respuestas)

Ausencia de felicidad (1) a felicidad extrema (5):

Ausencia de tristeza (1) a tristeza extremo (5):

Ausencia de enojo (1) a enojo extremo (5):

Ausencia de sorpresa (1) a sorpresa extremo (5):

Ausencia de asco (1) a asco extremo (5):

---

Sección F

En cada uno de los siguientes enunciados se le pide que se imagine sintiéndose de una manera determinada. Conteste lo mejor que pueda, incluso si no es capaz de imaginar ese sentimiento. Responda de acuerdo a la siguiente escala: **Nada parecido (1) Muy parecido (5)**

---

**F\_1**

Imagine que se siente culpable porque olvidó visitar a un buen amigo que tiene una enfermedad grave. Al mediodía se da cuenta de que ha olvidado por completo ir a verlo al hospital. ¿En qué grado ese sentimiento de culpabilidad se parece a cada uno de los siguientes términos?:

Frío

Azul

Dulce

---

**F\_2**

Imagínese que se siente contento en un día maravilloso y le va todo muy bien en el trabajo y con su Familia. ¿En qué grado ese sentimiento de satisfacción se parece a cada una de las siguientes sensaciones?:

Calido

Púrpura

Salado

---

**F\_3**

Imagine que se está sintiendo frío, lento y afilado. ¿En qué grado ese sentimiento se parece a cada uno de los siguientes términos?:

Desafiado

Aislado

Sorprendido

---

**F\_4**

Imagine que se siente intenso, grande, delicado y verde brillante. ¿En qué grado ese sentimiento se parece a cada uno de los siguientes términos?:

Entusiasmado

Celoso

Asustado

---

**F\_5**

Imagine que se siente cerrado, oscuro y paralizado. ¿En qué grado ese sentimiento se parece a cada uno de los siguientes términos?:

Triste

Contento

Calmado

---

Sección G

Seleccione la mejor alternativa en cada uno de los siguientes enunciados.

---

**G\_1**

Un sentimiento de preocupación es la combinación de emociones de: **1. amor, ansiedad, sorpresa y enojo; 2. sorpresa, orgullo, enojo y miedo; 3. aceptación, ansiedad, miedo y expectación; 4. miedo, alegría, sorpresa y verguenza; 5. ansiedad, cuidado y expectación**

---

**G\_2**

Otra palabra para «esperar sistematicamente el placer» es: **1. optimismo; 2. felicidad; 3. satisfacción; 4. alegría; 5. sorpresa**

---

**G\_3**

Aceptación, alegría y afecto se combinan con frecuencia para formar: **1. amor; 2. asombro; 3. expectación; 4. satisfacción; 5. aceptación**

---

**G\_4**

Combinar los sentimientos de asco y enojo da como resultado: **1. culpa; 2. rabia; 3. verguenza; 4. odio; 5. desprecio**

---

**G\_5**

Una sorpresa triste lleva a: **1. la desilusión; 2. el asombro; 3. el enojo; 4. el miedo; 5. el arrepentimiento**

---

**G\_6**

Tristeza, culpa y arrepentimiento se combinan para formar: **1. dolor; 2. enfado; 3. depresión; 4. remordimiento; 5. miseria**

---

**G\_7**

Relajación, seguridad y serenidad son parte de: **1. el amor; 2. la fatiga; 3. la expectación; 4. la calma; 5. la anticipación**

---

**G\_8**

Miedo, alegría, sorpresa y verguenza son parte de: **1. el aprecio; 2. el sobrecogimiento; 3. la perplejidad; 4. el respeto; 5. la comprensión**

---

**G\_9**

Verguenza, sorpresa y bochorno están combinados en el sentimiento de: **1. celos; 2. tristeza; 3. culpa; 4. envidia; 5. humillación**

---

**G\_10**

Admiracion, amor y ansiedad son parte de: **1. los celos; 2. la tristeza; 3. la maldad; 4. el orgullo; 5. la preocupación**

---

**G\_11**

Alegría, entusiasmo e incertidumbre son parte del sentimiento de: **1. vivacidad; 2. expectación; 3. ansiedad; 4. calma; 5. serenidad**

---

**G\_12**

Tristeza y satisfacci6n son a veces parte del sentimiento de: **1. nostalgia; 2. ansiedad; 3. expectación; 4. depresión; 5. desprecio**

---

Sección H

Seleccione el grado de eficacia de cada una de las siguientes respuestas y márquela.

---

**H\_1**

El año pasado Juan hizo un buen amigo en el trabajo. Hoy, ese amigo le ha sorprendido por completo al decirle que había aceptado un trabajo en otra compañía y que se mudaría de esa zona. No le había dicho nada a Juan de que estuviera buscando otros trabajos. En qué grado sería efectivo para Juan, con la finalidad de mantener la amistad, responder de la siguiente manera?:

Respuesta 1: Se sintió feliz por su amigo y le dijo que estaba muy contento de que consiguiera otro nuevo trabajo. A partir de ahora, Juan se aseguraría de que mantuvieran el contacto (**1. Muy ineficaz 2. Algo ineficaz 3. Ni eficaz, ni ineficaz 4. Algo eficaz 5. Muy eficaz**)

Respuesta 2: Juan se sintió triste porque su amigo iba a marcharse, consideraba que lo que había ocurrido era una muestra de que no le importaba a su amigo. Después de todo, su amigo no le había comentado que estuviera buscando otro empleo. Ya que se marcharía de todos modos, Juan no le dijo nada y empezó a buscar nuevos amigos en el trabajo (**1. Muy ineficaz 2. Algo ineficaz 3. Ni eficaz, ni ineficaz 4. Algo eficaz 5. Muy eficaz**)

Respuesta 3: Juan estaba muy enojado porque su amigo no le había dicho nada y mostró su desaprobación ignorándolo hasta que le diera una explicación. Juan pensó que si no le decía nada, eso confirmaría su opinión de que no valía la pena hablar con él (**1. Muy ineficaz 2. Algo ineficaz 3. Ni eficaz, ni ineficaz 4. Algo eficaz 5. Muy eficaz**)

---

**H\_2**

La profesora de Raúl acaba de llamar a sus padres para decirles que va muy mal en el colegio. La profesora les dice que su hijo no pone atención, es problemático y no puede estarse quieto. Esta profesora en particular no se maneja muy bien con los chicos inquietos y los padres de Raúl se preguntan qué esta pasando realmente. Más tarde, la profesora les dice que su hijo volverá a repetir curso a menos que mejore. Los padres se sienten muy enojados. ¿En qué grado sera útil para su hijo cada una de estas reacciones?:

Respuesta 1: Los padres le dijeron a la profesora que eso supone una gran conmoción para ellos y que era la primera vez que escuchaban que existiese ese problema. Piden reunirse con la profesora y solicitan que el director también asista a la reunión (**1. Muy ineficaz 2. Algo ineficaz 3. Ni eficaz, ni ineficaz 4. Algo eficaz 5. Muy eficaz**)

Respuesta 2: Los padres le dijeron a la profesora que si ella continuaba amenazando con que su hijo repitiera el curso, llevarían este problema al director. Le dijeron: «Si nuestro hijo repite, diremos que usted es personalmente la responsable. Usted es la profesora y su trabajo es enseñar, no echarle la culpa a los alumnos» (**1. Muy ineficaz 2. Algo ineficaz 3. Ni eficaz, ni ineficaz 4. Algo eficaz 5. Muy eficaz**)

Respuesta 3: Los padres de Raúl le colgaron el teléfono a la profesora y llamaron al director. Se quejaron de las amenazas de la profesora y solicitaron que su hijo fuera trasladado a una clase diferente (**1. Muy ineficaz 2. Algo ineficaz 3. Ni eficaz, ni ineficaz 4. Algo eficaz 5. Muy eficaz**)

---

**H\_3**

A Lisa le va todo bien. Mientras los demás han recibido críticas por su trabajo, Lisa ha conseguido un ascenso y un aumento de sueldo bastante bueno. Sus hijos están muy contentos y van muy bien en el colegio, su matrimonio es estable y muy feliz. Lisa empieza a sentirse muy orgullosa de sí misma y siente la tentación de presumir sobre su vida ante sus amigos. ¿En qué medida serían eficaces cada una de las siguientes respuestas para mantener sus relaciones?:

Respuesta 1: Puesto que todo va tan bien es bueno sentirse orgullosa. Pero Lisa también se dio cuenta de que algunas personas ven esto como una actitud arrogante o pueden sentirse celosas y por tanto sólo expresó sus sentimientos a amigos muy cercanos (**1. Muy ineficaz 2. Algo ineficaz 3. Ni eficaz, ni ineficaz 4. Algo eficaz 5. Muy eficaz**)

Respuesta 2: Lisa pensó en todas las cosas que podrían ir mal en el futuro, así tendría una visión más completa de su vida. Comprendió que los sentimientos positivos no duran siempre (**1. Muy ineficaz 2. Algo ineficaz 3. Ni eficaz, ni ineficaz 4. Algo eficaz 5. Muy eficaz**)

Respuesta 3: Esa noche Lisa compartió sus sentimientos con su marido. Poco después, decidió que la familia debería pasar más tiempo junta los fines de semana y hacer más actividades familiares para estar unidos (**1. Muy ineficaz 2. Algo ineficaz 3. Ni eficaz, ni ineficaz 4. Algo eficaz 5. Muy eficaz**)


---

Fin de la prueba. Compruebe que ha respondido a todas las preguntas

---

Aceptar
Cancelar
