# Supplementary material for: CuentosIE: can a chatbot about “tales with a message” help to teach emotional intelligence?
Source: PeerJ Comput Sci. 2024 Feb 29;10:e1866. doi: 10.7717/peerj-cs.1866 (PMC10909183; doi:10.7717/peerj-cs.1866)
Supplement: Supplemental Information 4 [file peerj-cs-10-1866-s004.tgz › testMillon.php]

CuentosIE: chatbot de Cuentos con mensaje para aprender Inteligencia Emocional


Tecnologías 

Configuración 
Añadir cuentos*note\_add*


Ayuda *help\_outline*
Contacto 
Usuarios 
Fin *call\_end*

##### *Usuario no registrado*

*live\_help*


#### MAC1. Test Millon

×

Esta prueba consiste en una lista de frases que la gente joven usa para describirse a sí misma. Se presentan aquí para ayudarte a describir tus sentimientos y actitudes. Cuando contestes trata de hacerlo tan honesta y seriamente como puedas, ya que los resultados serán utilizados para ayudar a conocerte y poder ayudarte a planear tu futuro. No te preocupes si algunas de las frases no te parecen muy corrientes.

Trata de responder a todas las frases aun cuando no estés seguro de tu decisión.

No hay límite de tiempo, aunque es mejor trabajar a un ritmo rápido pero cómodo.

***LOS RESULTADOS DE ESTA PRUEBA SON CONFIDENCIALES***

---

**Nombre de usuario en CuentosIE:**

---

**P1** Yo prefiero mucho más seguir a otro en vez de mandar.

Verdadero

Falso

---

**P2** Estoy bastante seguro de que sé quién soy y de lo que quiero en la vida.

Verdadero

Falso

---

**P3** Yo no necesito tener amistades íntimas como los otros jóvenes.

Verdadero

Falso

---

**P4** A menudo me fastidia hacer las cosas que los otros quieren que haga.

Verdadero

Falso

---

**P5** Me esfuerzo al máximo para no herir los sentimientos de otras personas.

Verdadero

Falso

---

**P6** Puedo confiar en que mis padres serán comprensivos conmigo.

Verdadero

Falso

---

**P7** Algunas personas piensan de mí que soy un poco creído.

Verdadero

Falso

---

**P8** Nunca tomaría drogas, pasase lo que pasase.

Verdadero

Falso

---

**P9** Siempre trato de hacer lo que es adecuado.

Verdadero

Falso

---

**P10** Me gusta mi aspecto.

Verdadero

Falso

---

**P11** Aunque a veces me descontrolo comiendo, odio cuando aumento de peso.

Verdadero

Falso

---

**P12** Parece que nada de lo que pasa me hace sentir ni triste ni feliz.

Verdadero

Falso

---

**P13** Parece que tengo problemas para llevarme bien con otros adolescentes de mi edad.

Verdadero

Falso

---

**P14** Me da mucha vergüenza contarle a otras personas cómo abusaron de mí.

Verdadero

Falso

---

**P15** Nunca he hecho nada por lo que hubiera podido ser arrestado.

Verdadero

Falso

---

**P16** Pienso que todos estarían mejor si yo estuviera muerto.

Verdadero

Falso

---

**P17** A veces, cuando estoy lejos de mi casa, empiezo a sentirme tenso y con miedo.

Verdadero

Falso

---

**P18** Generalmente actúo de forma rápida, sin pensar.

Verdadero

Falso

---

**P19** Supongo que soy un quejica que espera que pase lo peor.

Verdadero

Falso

---

**P20** No es raro sentirse solo y no deseado.

Verdadero

Falso

---

**P21** El castigo nunca me ha frenado para hacer lo que yo quería.

Verdadero

Falso

---

**P22** Parece ser que la bebida ha sido un problema para varios miembros de mi familia.

Verdadero

Falso

---

**P23** Me gusta seguir instrucciones y hacer lo que otros esperan de mí.

Verdadero

Falso

---

**P24** Me parece que encajo enseguida en cualquier grupo de chicos o chicas.

Verdadero

Falso

---

**P25** Muy poco de lo que he hecho ha sido apreciado por los demás.

Verdadero

Falso

---

**P26** Odio no tener ni el aspecto ni la inteligencia que quisiera tener.

Verdadero

Falso

---

**P27** Me gusta mi hogar.

Verdadero

Falso

---

**P28** Algunas veces meto miedo a otros chicos o chicas para que hagan lo que yo quiero.

Verdadero

Falso

---

**P29** Aunque la gente me dice que estoy delgado o delgada, yo sigo creyendo que peso demasiado .

Verdadero

Falso

---

**P30** Cuando tomo unas copas me siento más seguro de mí mismo.

Verdadero

Falso

---

**P31** La mayoría de la gente es más atractiva que yo.

Verdadero

Falso

---

**P32** Cuando estoy en medio de una multitud, a menudo siento que me va a dar un ataque de pánico o que me voy a desmayar.

Verdadero

Falso

---

**P33** A veces me provoco el vómito después de comer mucho.

Verdadero

Falso

---

**P34** Con frecuencia me siento como si no tuviera rumbo, como perdido en la vida.

Verdadero

Falso

---

**P35** Parece que no les caigo bien a la mayoría de los jóvenes.

Verdadero

Falso

---

**P36** Cuando puedo elegir, prefiero hacer las cosas solo o sola.

Verdadero

Falso

---

**P37** Implicarse en los problemas de otros es una pérdida de tiempo.

Verdadero

Falso

---

**P38** Muchas veces siento que otros no quieren ser amistosos conmigo.

Verdadero

Falso

---

**P39** No me importa mucho lo que otros jóvenes piensan de mí.

Verdadero

Falso

---

**P40** Solía «Colocarme» tanto (con alcohol o drogas) que no sabía lo que estaba haciendo.

Verdadero

Falso

---

**P41** No me importa decirle a la gente cosas que no le gusta oír.

Verdadero

Falso

---

**P42** Me veo a mí mismo muy lejos de lo que en realidad me gustaría ser.

Verdadero

Falso

---

**P43** Las cosas en mi vida van de mal en peor.

Verdadero

Falso

---

**P44** Tan pronto como tengo el impulso de hacer algo, lo hago.

Verdadero

Falso

---

**P45** A mí nunca me han llamado «delincuente juvenil».

Verdadero

Falso

---

**P46** Con frecuencia yo soy mi peor enemigo.

Verdadero

Falso

---

**P47** Muy pocas cosas o actividades parecen darme placer.

Verdadero

Falso

---

**P48** Siempre pienso en ponerme a dieta, aun cuando la gente me dice que estoy demasiado delgado o delgada.

Verdadero

Falso

---

**P49** Es muy difícil, para mí, sentir lástima por las personas que siempre están preocupadas por alguna cosa.

Verdadero

Falso

---

**P50** Es bueno tener una rutina para hacer la mayoría de las cosas.

Verdadero

Falso

---

**P51** No creo tener tanto interés por el sexo como la gente de mi edad.

Verdadero

Falso

---

**P52** No veo nada malo en utilizar a otros para conseguir lo que quiero.

Verdadero

Falso

---

**P53** Preferiría estar en cualquier lugar en vez de en casa.

Verdadero

Falso

---

**P54** A veces me siento tan desbordado que querría dañarme gravemente.

Verdadero

Falso

---

**P55** No creo haber sido abusado sexualmente.

Verdadero

Falso

---

**P56** Soy un tipo de persona teatral a quien le gusta llamar la atención.

Verdadero

Falso

---

**P57** Puedo beber más cerveza o licor que la mayoría de mis amigos.

Verdadero

Falso

---

**P58** Los padres y maestros son demasiado duros con los jóvenes que no siguen las reglas.

Verdadero

Falso

---

**P59** Me gusta mucho coquetear (ligar).

Verdadero

Falso

---

**P60** No me molesta ver a alguien sufriendo.

Verdadero

Falso

---

**P61** Al parecer no tengo muchos sentimientos por los demás.

Verdadero

Falso

---

**P62** Disfruto pensando en el sexo.

Verdadero

Falso

---

**P63** Me preocupa mucho pensar en que me dejen solo y abandonado.

Verdadero

Falso

---

**P64** Con frecuencia estoy triste y siento que nadie me quiere.

Verdadero

Falso

---

**P65** Se supone que debo ser delgado o delgada, pero siento que mis muslos y mi trasero son demasiado grandes.

Verdadero

Falso

---

**P66** Con frecuencia me lo merezco cuando los otros me critican.

Verdadero

Falso

---

**P67** La gente me presiona para que haga más de lo que es justo.

Verdadero

Falso

---

**P68** Creo que tengo un buen cuerpo.

Verdadero

Falso

---

**P69** Socialmente , siento que me dejan de lado.

Verdadero

Falso

---

**P70** Hago amigos y amigas fácilmente.

Verdadero

Falso

---

**P71** Soy una persona un tanto asustadiza y ansiosa.

Verdadero

Falso

---

**P72** Odio recordar algunas de las formas en que abusaron de mí.

Verdadero

Falso

---

**P73** No soy diferente a muchos jóvenes que roban cosas de vez en cuando.

Verdadero

Falso

---

**P74** Prefiero actuar primero y pensarlo después.

Verdadero

Falso

---

**P75** He pasado por periodos en los que he fumado canutos varias veces a la semana.

Verdadero

Falso

---

**P76** Hay tantas normas en mi camino, que es difícil hacer lo que quiero.

Verdadero

Falso

---

**P77** Cuando las cosas se ponen aburridas, me gusta crear un poco de emoción.

Verdadero

Falso

---

**P78** A veces yo haría algo cruel para hacer infeliz a alguien.

Verdadero

Falso

---

**P79** Paso mucho tiempo preocupándome acerca de mi futuro.

Verdadero

Falso

---

**P80** Con frecuencia siento que no me merezco las cosas buenas que hay en mi vida.

Verdadero

Falso

---

**P81** Me da un poco de tristeza cuando veo a alguien que se siente solo.

Verdadero

Falso

---

**P82** Como poco delante de otros, pero después me atiborro cuando estoy solo o sola.

Verdadero

Falso

---

**P83** Mi familia siempre está gritando y peleándose.

Verdadero

Falso

---

**P84** A veces me siento muy infeliz de ser quien soy.

Verdadero

Falso

---

**P85** Al parecer yo no disfruto estando con gente.

Verdadero

Falso

---

**P86** Tengo ciertas capacidades que otros jóvenes quisieran tener.

Verdadero

Falso

---

**P87** Me siento muy incómodo con la gente, a menos que esté seguro de que realmente les gusto.

Verdadero

Falso

---

**P88** Matarme sería la manera más fácil de resolver mis problemas.

Verdadero

Falso

---

**P89** A veces me confunde o me turba que la gente sea amable conmigo.

Verdadero

Falso

---

**P90** La bebida parece que me ayuda mucho cuando me siento deprimido.

Verdadero

Falso

---

**P91** Casi nunca espero algún acontecimiento con placer o emoción.

Verdadero

Falso

---

**P92** Soy muy bueno inventando excusas para salir de los problemas.

Verdadero

Falso

---

**P93** Es muy importante que los niños aprendan a obedecer a los mayores.

Verdadero

Falso

---

**P94** El sexo es algo placentero.

Verdadero

Falso

---

**P95** A nadie le importa realmente si yo vivo o si me muero.

Verdadero

Falso

---

**P96** Deberíamos respetar a nuestros mayores y no pensar que nosotros sabemos más.

Verdadero

Falso

---

**P97** A veces siento placer lastimando a alguien físicamente.

Verdadero

Falso

---

**P98** A veces me siento mal después de que me haya pasado algo bueno.

Verdadero

Falso

---

**P99** No creo que la gente me vea como una persona atractiva.

Verdadero

Falso

---

**P100** Socialmente soy solitario, pero no me importa.

Verdadero

Falso

---

**P101** Casi todo lo que intento me resulta fácil.

Verdadero

Falso

---

**P102** Hay veces en las que siento que soy mucho más joven de lo que realmente soy.

Verdadero

Falso

---

**P103** Me gusta ser el centro de atención.

Verdadero

Falso

---

**P104** Si quiero hacer algo, simplemente lo hago, sin pensar en lo que pueda pasar.

Verdadero

Falso

---

**P105** Tengo un temor terrible de que, por muy delgado o delgada que esté, volveré a subir de peso si como.

Verdadero

Falso

---

**P106** No me acerco mucho a las personas porque me da miedo que se burlen de mí.

Verdadero

Falso

---

**P107** Cada vez con más frecuencia he pensado en terminar con mi vida.

Verdadero

Falso

---

**P108** A veces me rebajo a mí mismo para que otra persona se sienta mejor.

Verdadero

Falso

---

**P109** Me da mucho miedo cuando pienso en estar completamente solo en el mundo.

Verdadero

Falso

---

**P110** Las cosas buenas no duran.

Verdadero

Falso

---

**P111** He tenido algunos choques con la ley.

Verdadero

Falso

---

**P112** Me gustaría cambiar mi cuerpo por el de alguna otra persona.

Verdadero

Falso

---

**P113** Hay muchas ocasiones en las que desearía ser mucho más joven de nuevo.

Verdadero

Falso

---

**P114** No he visto un automóvil en los últimos diez años.

Verdadero

Falso

---

**P115** Otras personas de mi edad parecen estar más seguras que yo de saber quiénes son y lo que quieren.

Verdadero

Falso

---

**P116** Muchas veces me desconcierta pensar en el sexo.

Verdadero

Falso

---

**P117** Hago lo que quiero sin preocuparme de si afecta a otros.

Verdadero

Falso

---

**P118** Muchas de las cosas que hoy parecen buenas, se volverán malas más adelante.

Verdadero

Falso

---

**P119** Parece que la gente de mi edad nunca me llama para que salgamos juntos.

Verdadero

Falso

---

**P120** Ha habido veces en las que no he podido pasar el día sin un «canuto».

Verdadero

Falso

---

**P121** Yo hago que mi vida sea peor de lo que debería ser.

Verdadero

Falso

---

**P122** Prefiero que me digan lo que tengo que hacer en lugar de tener que decidir por mí mismo.

Verdadero

Falso

---

**P123** He intentado suicidarme, en el pasado.

Verdadero

Falso

---

**P124** Me pego grandes atracones de comida un par de veces a la semana.

Verdadero

Falso

---

**P125** Últimamente parece que me deprimo por cosas pequeñas.

Verdadero

Falso

---

**P126** El año pasado crucé el Atlántico en avión 30 veces.

Verdadero

Falso

---

**P127** Hay veces en las que preferiría ser otra persona.

Verdadero

Falso

---

**P128** No me importa tratar mal a la gente para demostrar mi poder.

Verdadero

Falso

---

**P129** Me avergüenzo de algunas cosas terribles que me han hecho los adultos.

Verdadero

Falso

---

**P130** Intento hacer todo lo que hago lo más perfectamente que puedo.

Verdadero

Falso

---

**P131** Estoy contento con la forma en que mi cuerpo se ha desarrollado.

Verdadero

Falso

---

**P132** Con frecuencia me asusto cuando pienso en las cosas que tengo que hacer.

Verdadero

Falso

---

**P133** Últimamente me siento inquieto y nervioso casi todo el tiempo.

Verdadero

Falso

---

**P134** Yo acostumbraba a probar drogas duras para ver el efecto que hacían.

Verdadero

Falso

---

**P135** Con mi simpatía puedo conseguir que la gente me dé casi todo lo que quiero.

Verdadero

Falso

---

**P136** Muchos otros jóvenes consiguen oportunidades que yo no consigo.

Verdadero

Falso

---

**P137** Hubo personas que hicieron cosas sexuales conmigo cuando yo,todavía, no podía entender.

Verdadero

Falso

---

**P138** Con frecuencia sigo comiendo hasta que me siento enfermo o enferma.

Verdadero

Falso

---

**P139** En un grupo, yo me burlaría de alguien sólo para humillarle.

Verdadero

Falso

---

**P140** No me gusta ser la persona en la que me he convertido.

Verdadero

Falso

---

**P141** Al parecer siempre echo a perder las cosas buenas que me pasan.

Verdadero

Falso

---

**P142** Aunque quiero tener amigos, no tengo casi ninguno.

Verdadero

Falso

---

**P143** Me agrada que ahora los sentimientos acerca del sexo se hayan convertido en una parte de mi vida.

Verdadero

Falso

---

**P144** Soy capaz de pasar mucha hambre con tal de ser más delgado o delgada de lo que soy.

Verdadero

Falso

---

**P145** Soy muy maduro para mi edad y sé lo que quiero hacer en la vida.

Verdadero

Falso

---

**P146** En muchas cosas me siento muy superior a la mayoría de la gente.

Verdadero

Falso

---

**P147** Mi futuro me parece sin esperanza.

Verdadero

Falso

---

**P148** A mis padres les ha costado mucho mantenerme en el camino recto.

Verdadero

Falso

---

**P149** Cuando no me salgo con la mía, me descontrolo fácilmente.

Verdadero

Falso

---

**P150** A veces me divierte hacer ciertas cosas que son ilegales.

Verdadero

Falso

---

**P151** Supongo que dependo demasiado de otros para que me ayuden.

Verdadero

Falso

---

**P152** Mis amigos y yo podemos acabar muy borrachos cuando lo estamos pasando bien.

Verdadero

Falso

---

**P153** La mayor parte del tiempo me siento solo y vacío.

Verdadero

Falso

---

**P154** Me siento totalmente sin objetivos y sin saber adónde voy.

Verdadero

Falso

---

**P155** Decir mentiras es una cosa muy normal.

Verdadero

Falso

---

**P156** He estado pensando en cómo y cuándo suicidarme.

Verdadero

Falso

---

**P157** Me gusta provocar peleas.

Verdadero

Falso

---

**P158** Hay veces en las que parece que no le importo a nadie en mi casa.

Verdadero

Falso

---

**P159** Es bueno tener una forma regular de hacer las cosas para no cometer errores.

Verdadero

Falso

---

**P160** Probablemente me merezco muchos de los problemas que tengo.

Verdadero

Falso


---

Aceptar
Cancelar
