# Supplementary figures and images for: CuentosIE: can a chatbot about “tales with a message” help to teach emotional intelligence?
Source: PeerJ Comput Sci. 2024 Feb 29;10:e1866. doi: 10.7717/peerj-cs.1866 (PMC10909183; doi:10.7717/peerj-cs.1866)

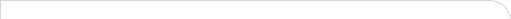

Supplement: Supplemental Information 4 [file peerj-cs-10-1866-s004.tgz › img/module_table_top.png]

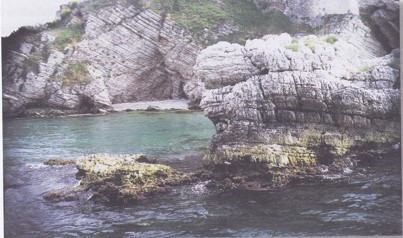

Supplement: Supplemental Information 4 [file peerj-cs-10-1866-s004.tgz › img/testMayerSalovey/E_1.jpg]

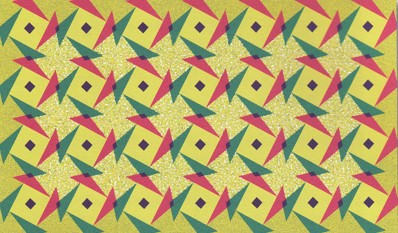

Supplement: Supplemental Information 4 [file peerj-cs-10-1866-s004.tgz › img/testMayerSalovey/E_5.jpg]

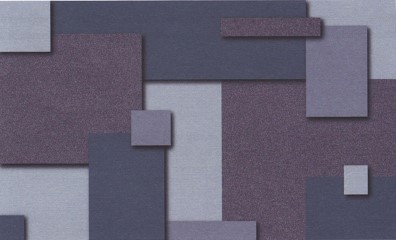

Supplement: Supplemental Information 4 [file peerj-cs-10-1866-s004.tgz › img/testMayerSalovey/E_6.jpg]

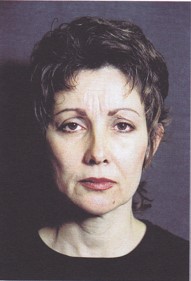

Supplement: Supplemental Information 4 [file peerj-cs-10-1866-s004.tgz › img/testMayerSalovey/A_3.jpg]

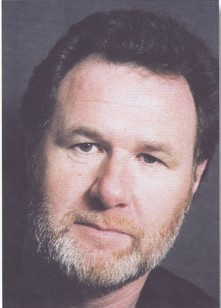

Supplement: Supplemental Information 4 [file peerj-cs-10-1866-s004.tgz › img/testMayerSalovey/A_1.jpg]

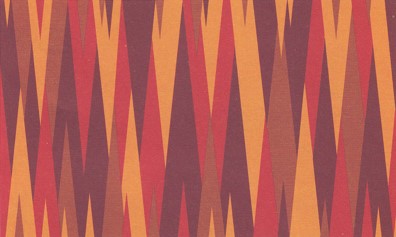

Supplement: Supplemental Information 4 [file peerj-cs-10-1866-s004.tgz › img/testMayerSalovey/E_4.jpg]

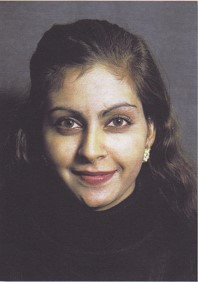

Supplement: Supplemental Information 4 [file peerj-cs-10-1866-s004.tgz › img/testMayerSalovey/A_4.jpg]

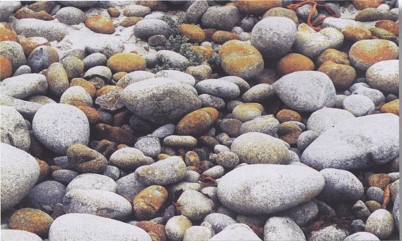

Supplement: Supplemental Information 4 [file peerj-cs-10-1866-s004.tgz › img/testMayerSalovey/E_3.jpg]

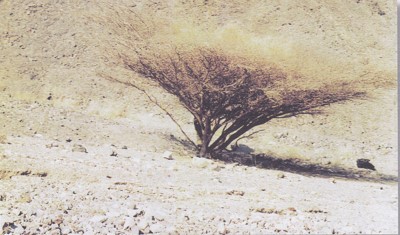

Supplement: Supplemental Information 4 [file peerj-cs-10-1866-s004.tgz › img/testMayerSalovey/E_2.jpg]

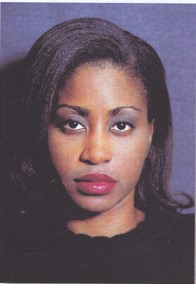

Supplement: Supplemental Information 4 [file peerj-cs-10-1866-s004.tgz › img/testMayerSalovey/A_2.jpg]

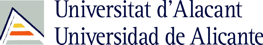

Supplement: Supplemental Information 4 [file peerj-cs-10-1866-s004.tgz › img/logos/logoua3.gif]

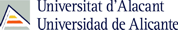

Supplement: Supplemental Information 4 [file peerj-cs-10-1866-s004.tgz › img/logos/logotipo-de-la-ua-178-x-28-pixel.gif]

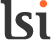

Supplement: Supplemental Information 4 [file peerj-cs-10-1866-s004.tgz › img/logos/logo_pla_petit.png]

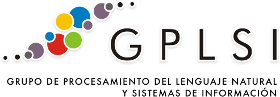

Supplement: Supplemental Information 4 [file peerj-cs-10-1866-s004.tgz › img/logos/gplsiweb.png]

Supplement: Supplemental Information 4 [file peerj-cs-10-1866-s004.tgz › img/logos/UAtransparente.gif]

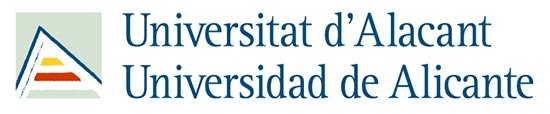

Supplement: Supplemental Information 4 [file peerj-cs-10-1866-s004.tgz › img/logos/logo-ua.jpg]

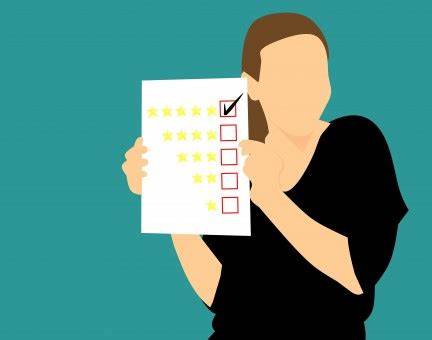

Supplement: Supplemental Information 4 [file peerj-cs-10-1866-s004.tgz › img/webBing/Recomendador.jfif]

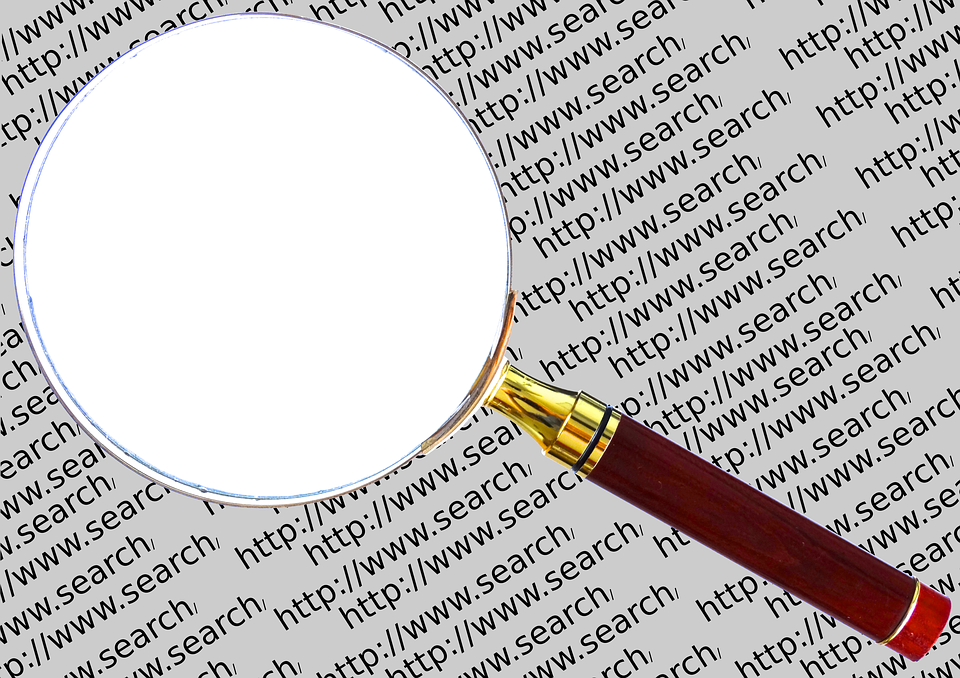

Supplement: Supplemental Information 4 [file peerj-cs-10-1866-s004.tgz › img/webBing/Buscador.png]

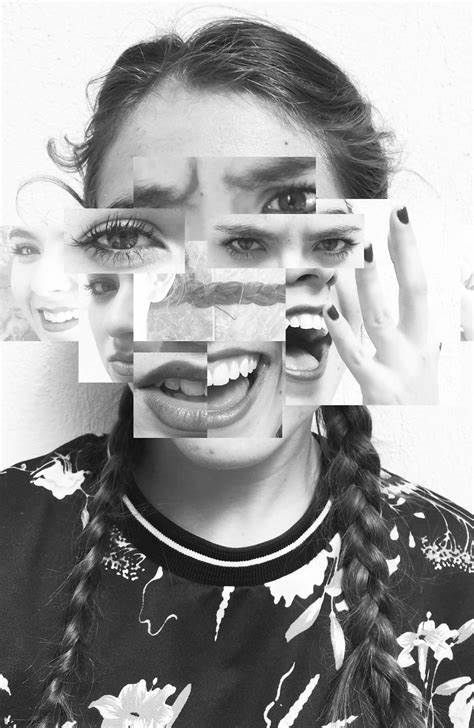

Supplement: Supplemental Information 4 [file peerj-cs-10-1866-s004.tgz › img/webBing/Emociones.jfif]

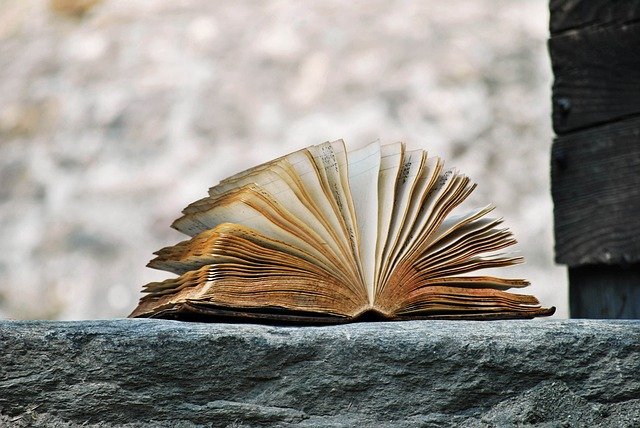

Supplement: Supplemental Information 4 [file peerj-cs-10-1866-s004.tgz › img/webBing/LibroAntiguoAbierto.jpg]

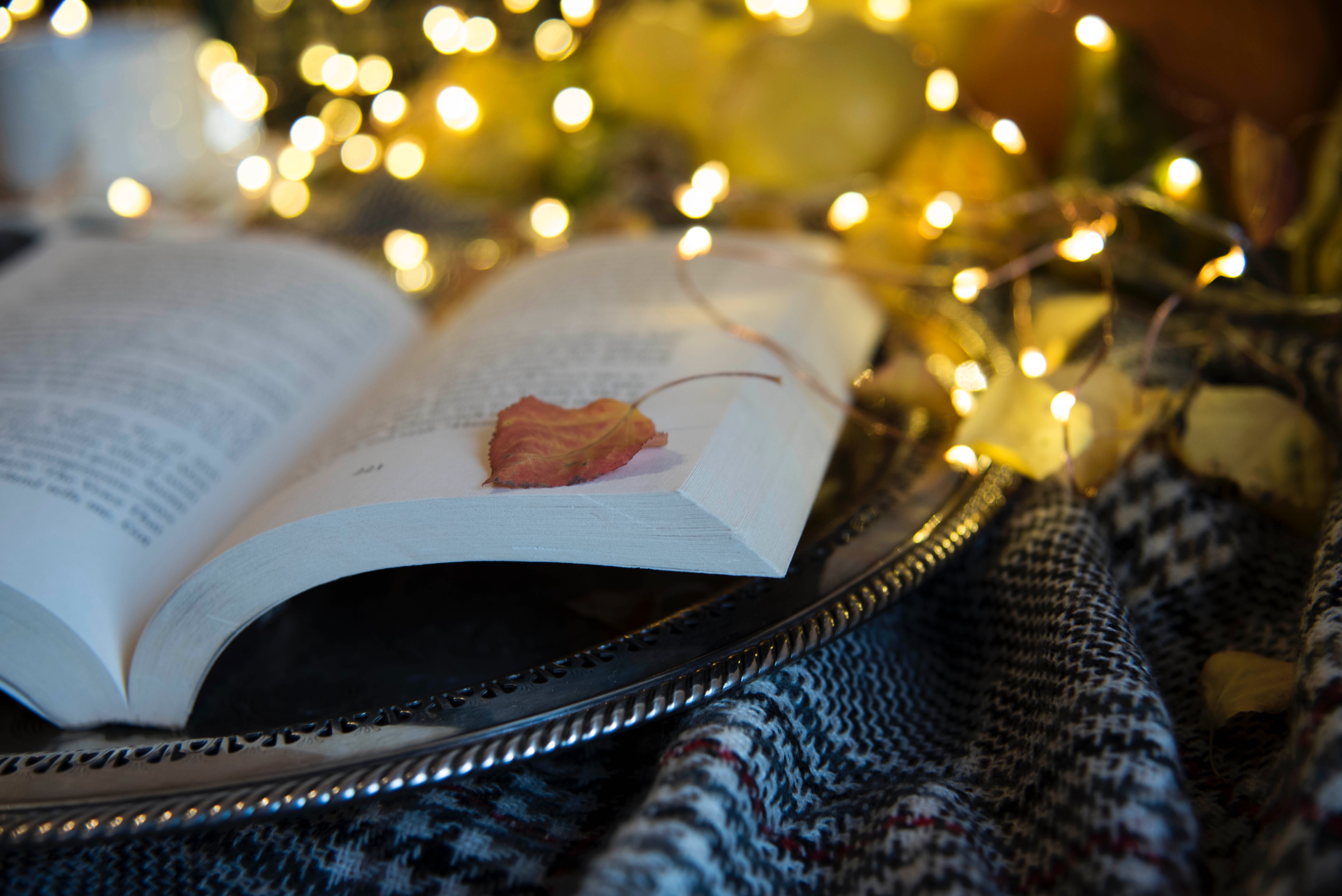

Supplement: Supplemental Information 4 [file peerj-cs-10-1866-s004.tgz › img/webBing/LibroLuces.jpg]

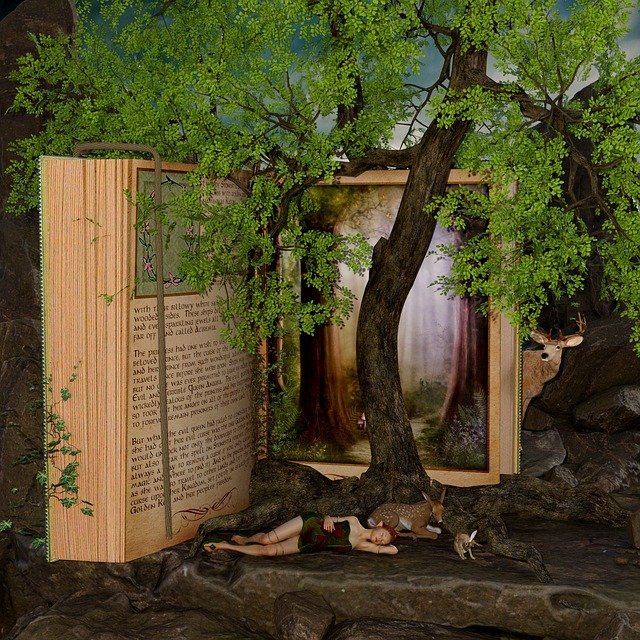

Supplement: Supplemental Information 4 [file peerj-cs-10-1866-s004.tgz › img/webBing/LibroArbol.jpg]

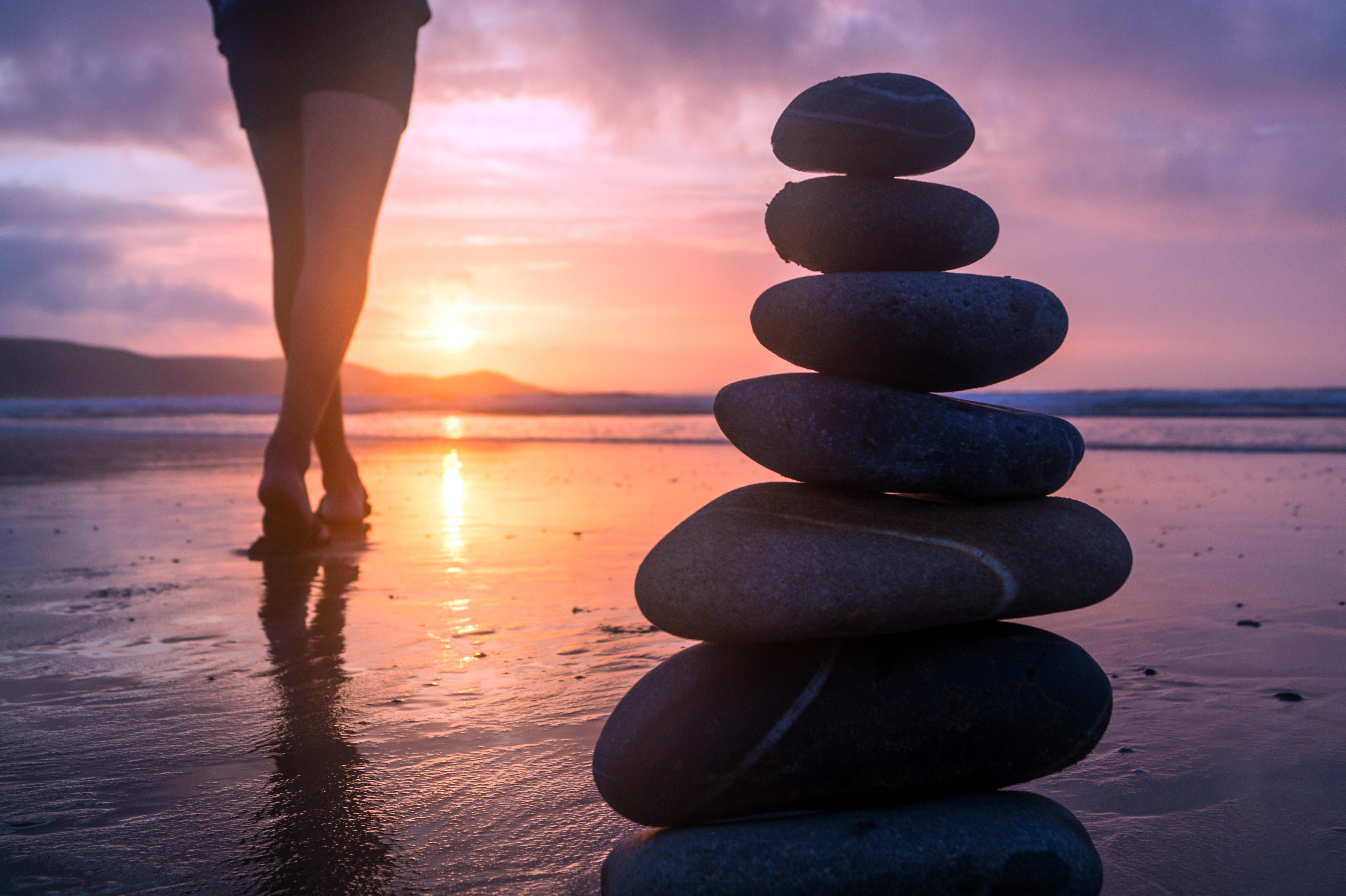

Supplement: Supplemental Information 4 [file peerj-cs-10-1866-s004.tgz › img/webBing/piedrasPlayaPies.jpg]

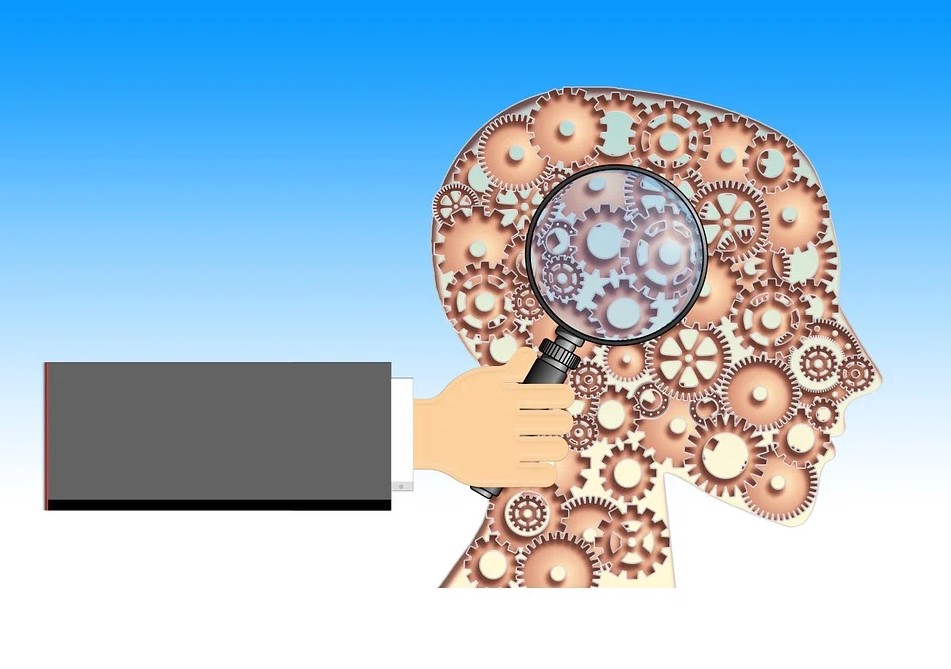

Supplement: Supplemental Information 4 [file peerj-cs-10-1866-s004.tgz › img/webBing/CerebroRuedasReloj.jpg]

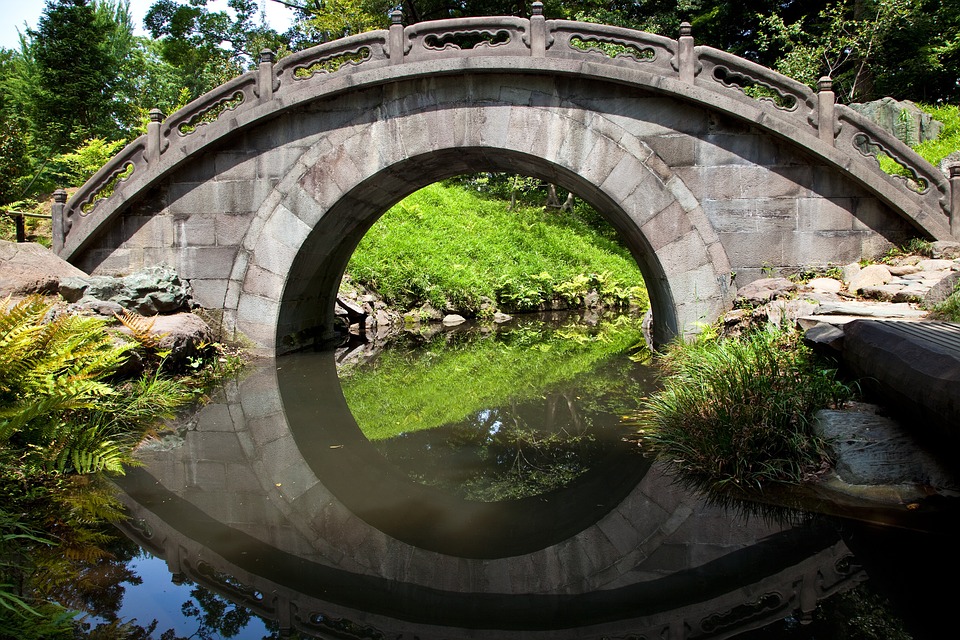

Supplement: Supplemental Information 4 [file peerj-cs-10-1866-s004.tgz › img/webBing/PuenteOjo.jpg]

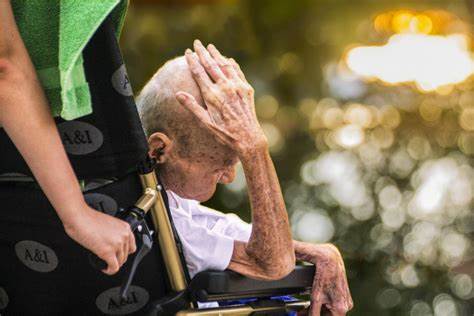

Supplement: Supplemental Information 4 [file peerj-cs-10-1866-s004.tgz › img/webBing/AncianoSillaRuedas.jfif]

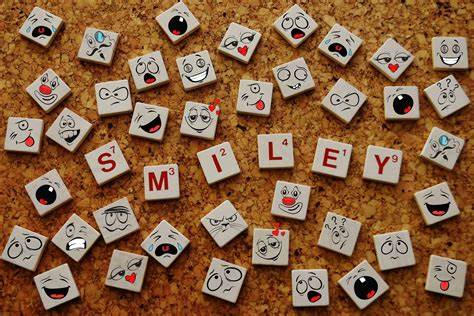

Supplement: Supplemental Information 4 [file peerj-cs-10-1866-s004.tgz › img/webBing/EmocionesLetras.jfif]

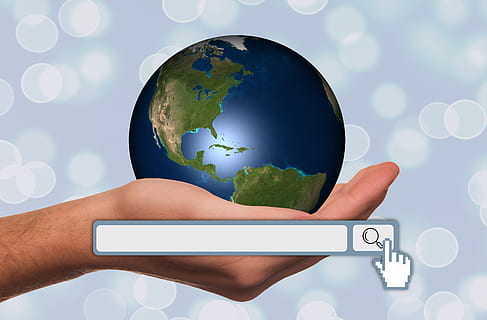

Supplement: Supplemental Information 4 [file peerj-cs-10-1866-s004.tgz › img/webBing/BuscadorManoMundo.jpg]

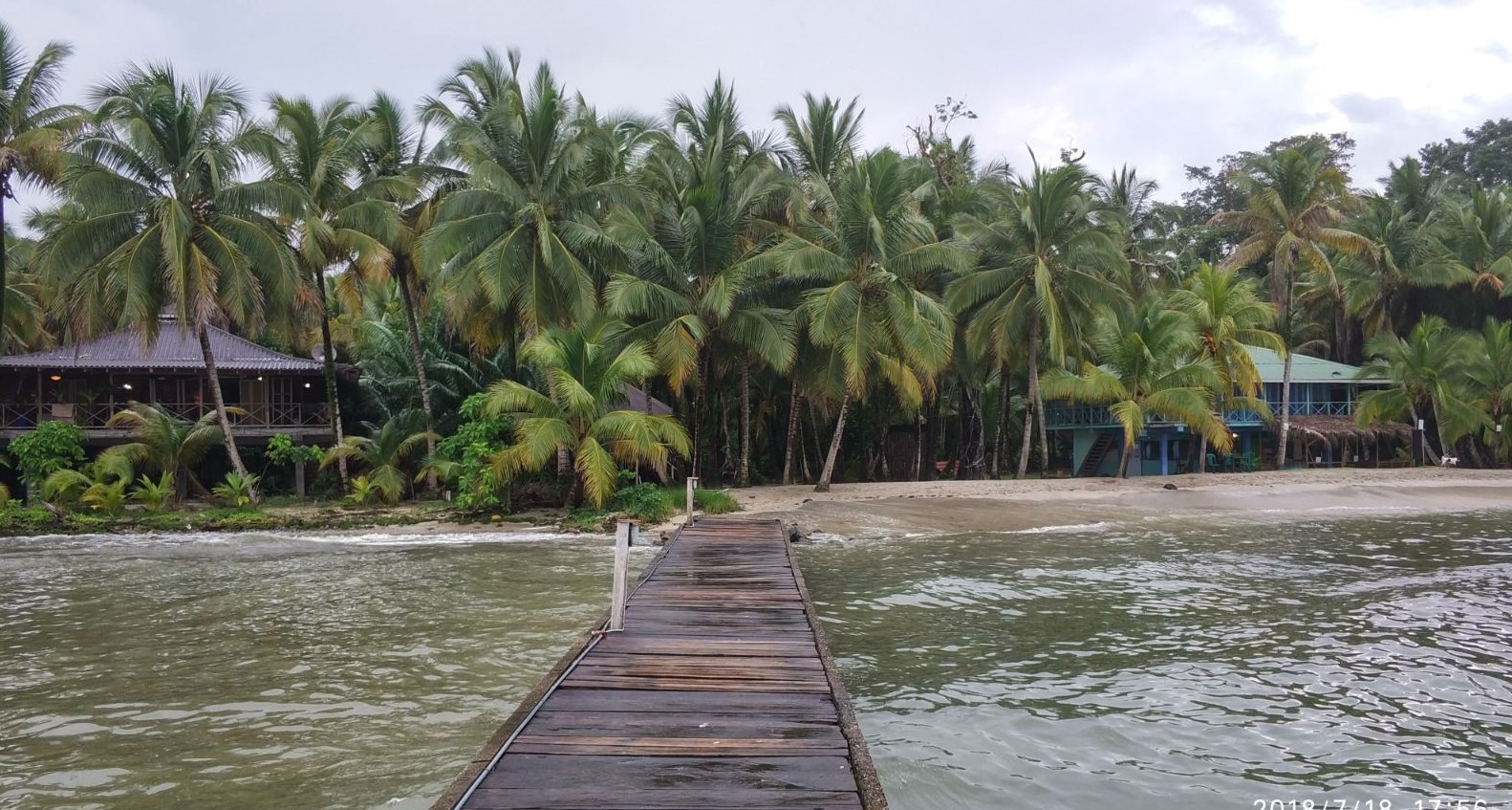

Supplement: Supplemental Information 4 [file peerj-cs-10-1866-s004.tgz › img/webBing/PalmerasPuente.jpg]

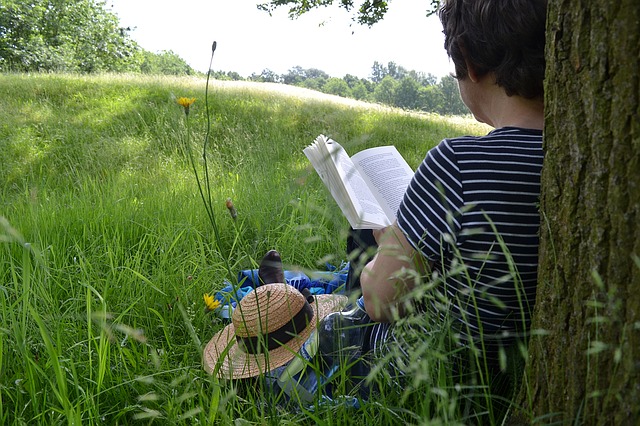

Supplement: Supplemental Information 4 [file peerj-cs-10-1866-s004.tgz › img/webBing/MujerLeyendoLibroArbol.jpg]

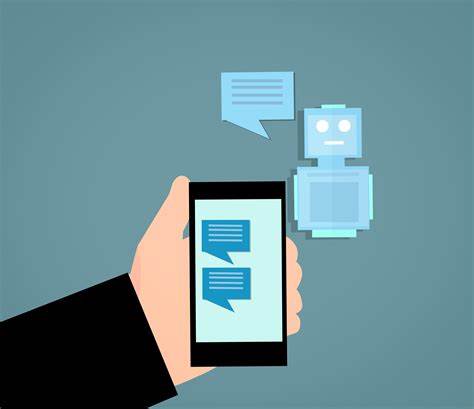

Supplement: Supplemental Information 4 [file peerj-cs-10-1866-s004.tgz › img/webBing/chatbot.jfif]

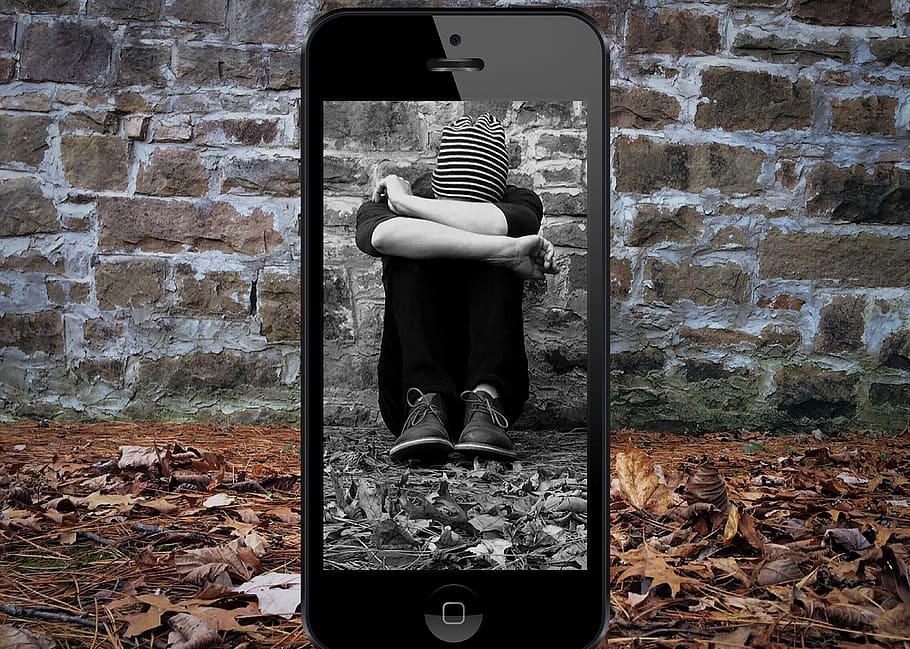

Supplement: Supplemental Information 4 [file peerj-cs-10-1866-s004.tgz › img/webBing/Bullying.jpg]

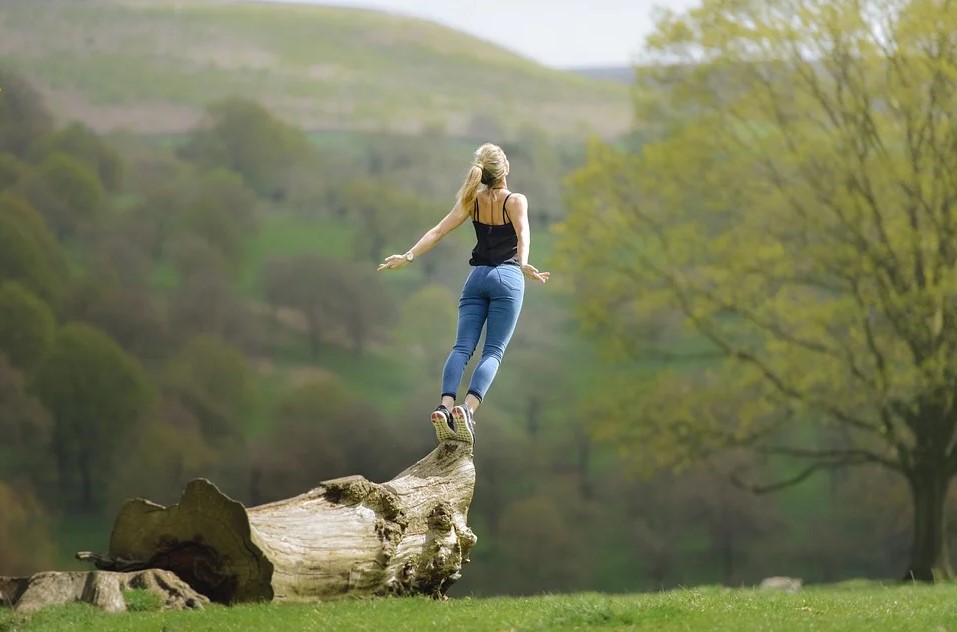

Supplement: Supplemental Information 4 [file peerj-cs-10-1866-s004.tgz › img/webBing/MujerTronco.jpg]

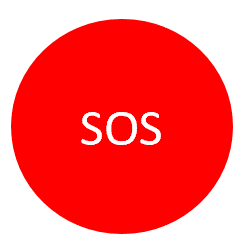

Supplement: Supplemental Information 4 [file peerj-cs-10-1866-s004.tgz › img/sos2.gif]

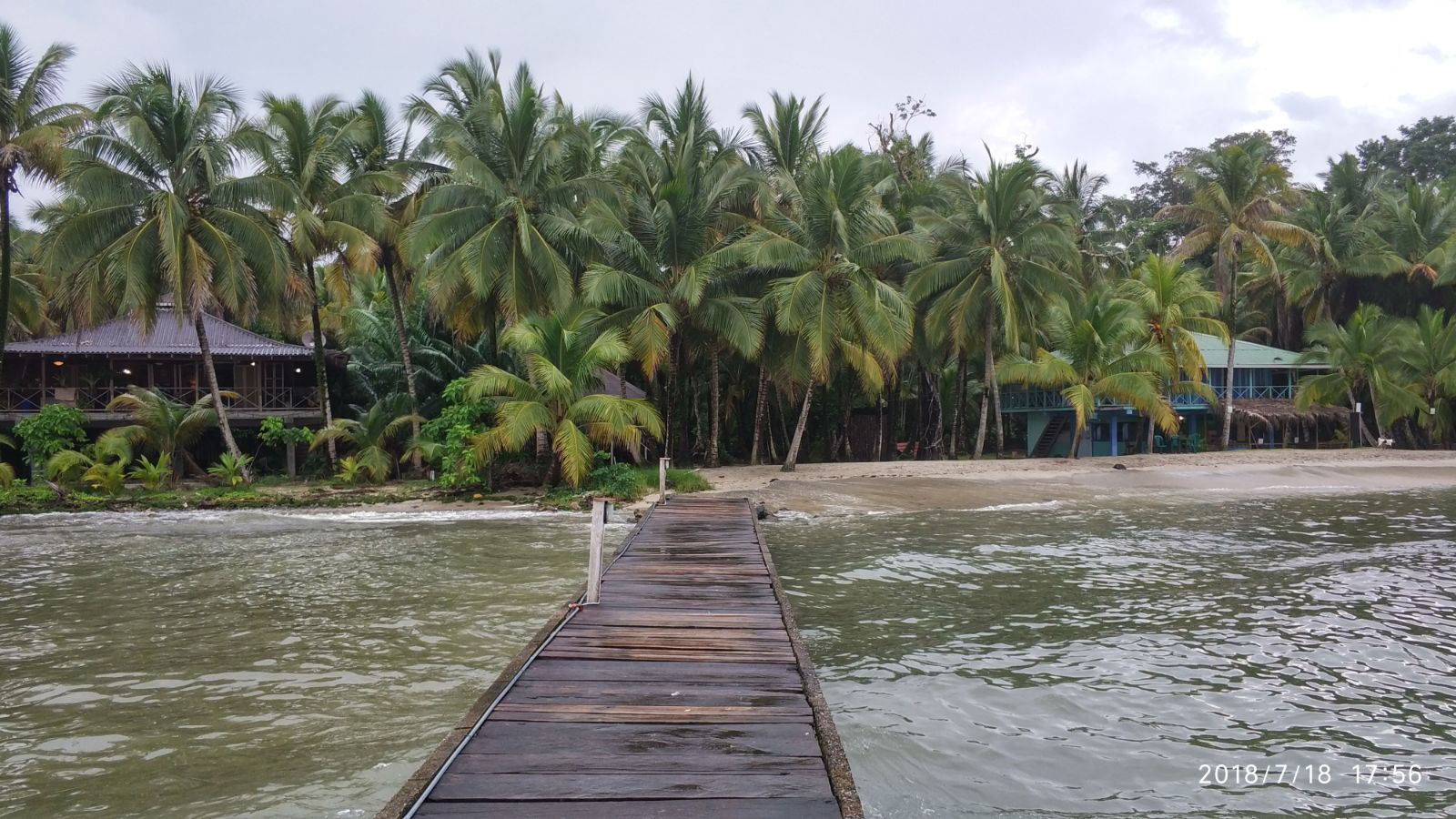

Supplement: Supplemental Information 4 [file peerj-cs-10-1866-s004.tgz › img/IMG-20200208-WA0001.jpg]

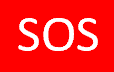

Supplement: Supplemental Information 4 [file peerj-cs-10-1866-s004.tgz › img/sos3.gif]

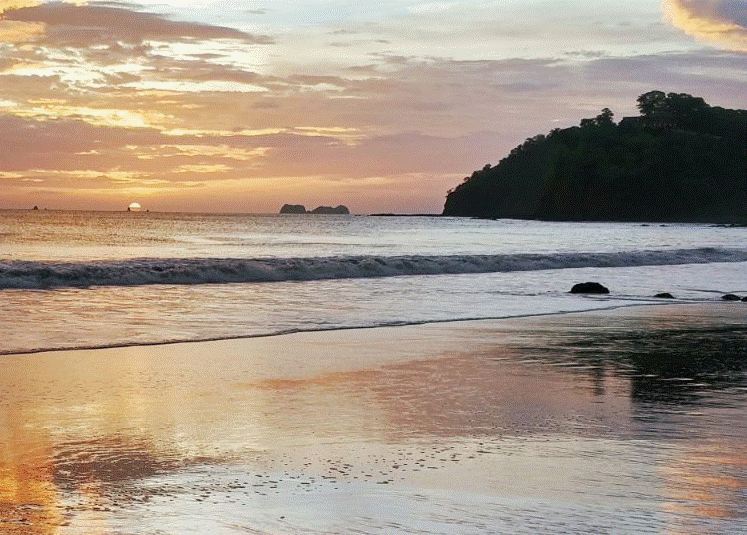

Supplement: Supplemental Information 4 [file peerj-cs-10-1866-s004.tgz › img/playa.gif]

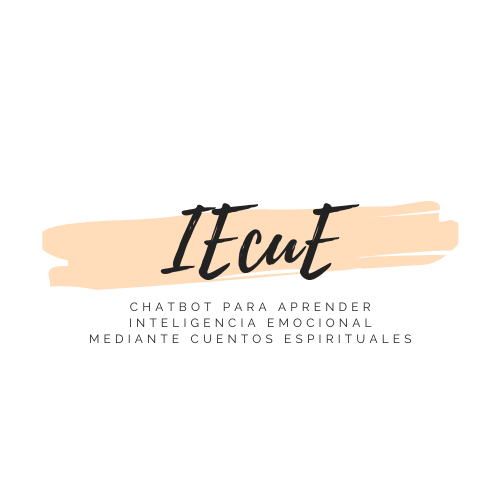

Supplement: Supplemental Information 4 [file peerj-cs-10-1866-s004.tgz › img/IEcuE.png]

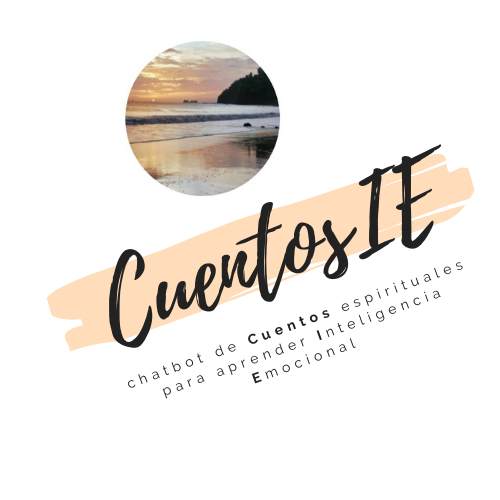

Supplement: Supplemental Information 4 [file peerj-cs-10-1866-s004.tgz › img/CuentosIE.png]

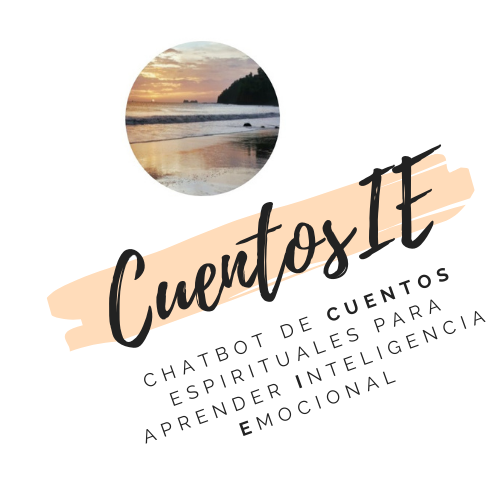

Supplement: Supplemental Information 4 [file peerj-cs-10-1866-s004.tgz › img/CuentosIE_2.png]

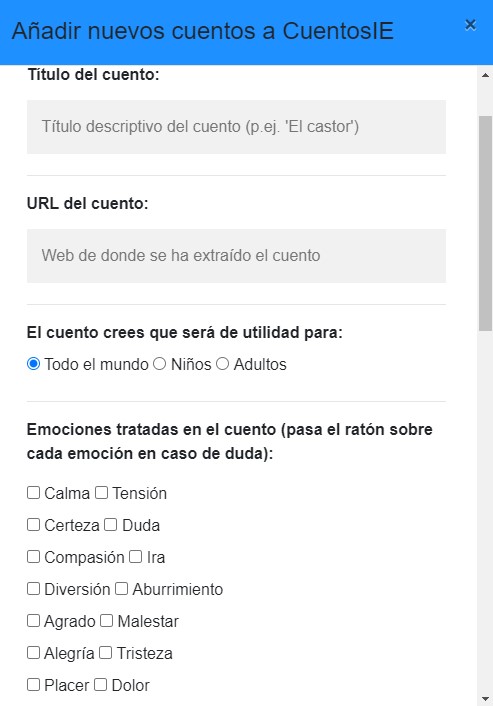

Supplement: Supplemental Information 4 [file peerj-cs-10-1866-s004.tgz › img/CuentosIE/IntroducirCuentos.jpg]

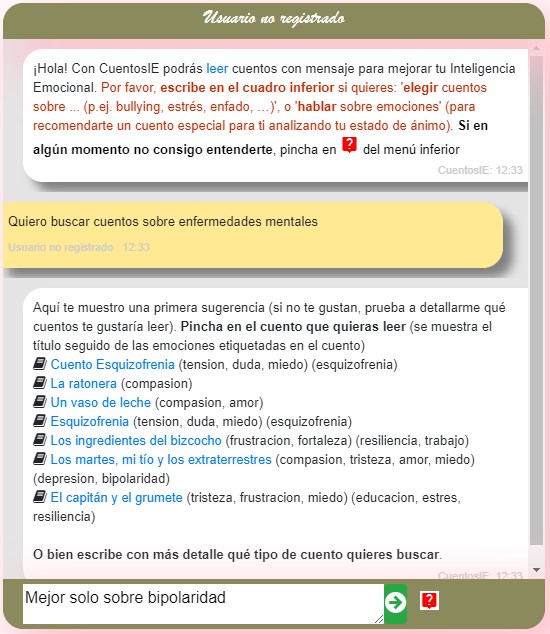

Supplement: Supplemental Information 4 [file peerj-cs-10-1866-s004.tgz › img/CuentosIE/Elegir2.jpg]

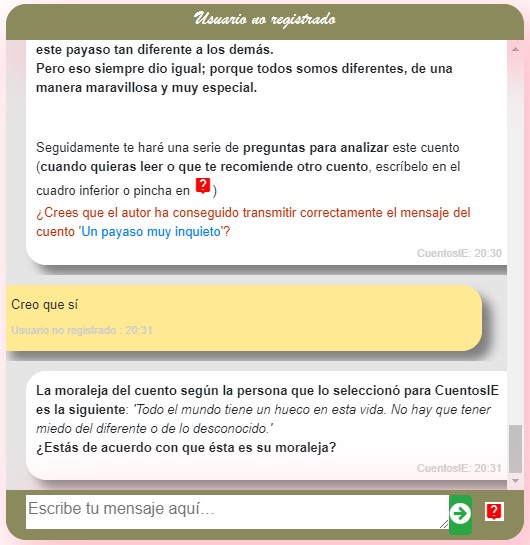

Supplement: Supplemental Information 4 [file peerj-cs-10-1866-s004.tgz › img/CuentosIE/Analisis1.jpg]

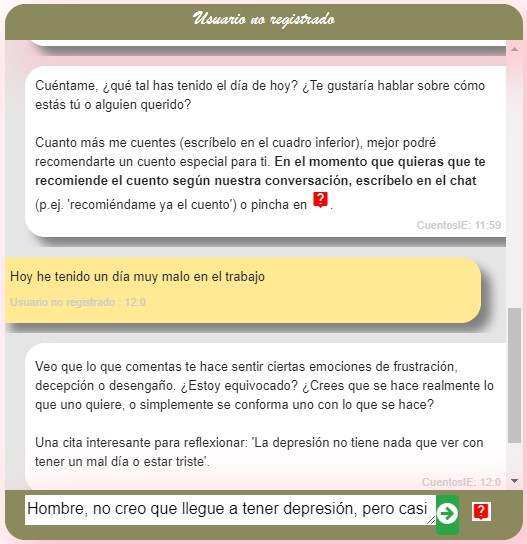

Supplement: Supplemental Information 4 [file peerj-cs-10-1866-s004.tgz › img/CuentosIE/Recomendar1.jpg]

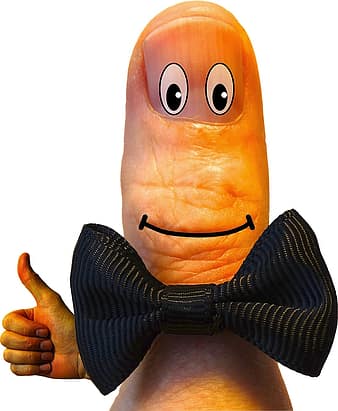

Supplement: Supplemental Information 4 [file peerj-cs-10-1866-s004.tgz › img/emociones/SATISFACCIÓN.jpg]

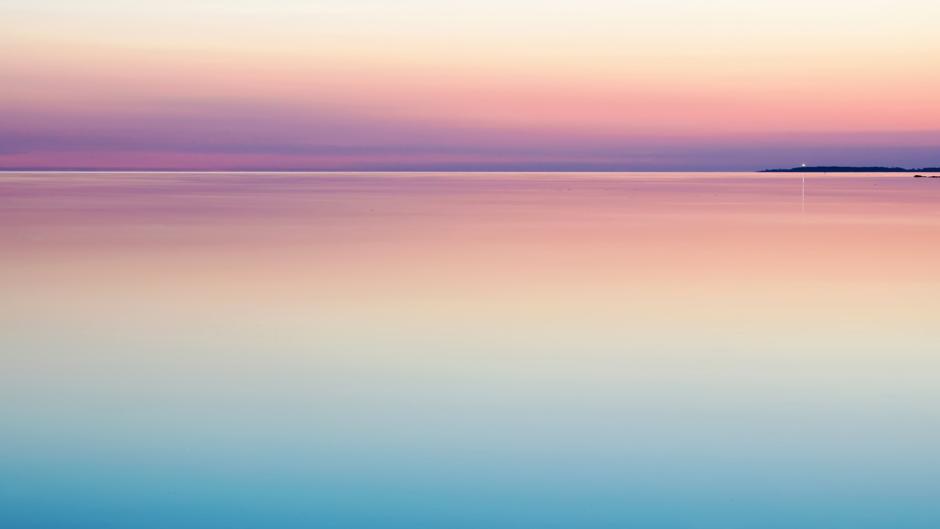

Supplement: Supplemental Information 4 [file peerj-cs-10-1866-s004.tgz › img/emociones/CALMA.jpg]

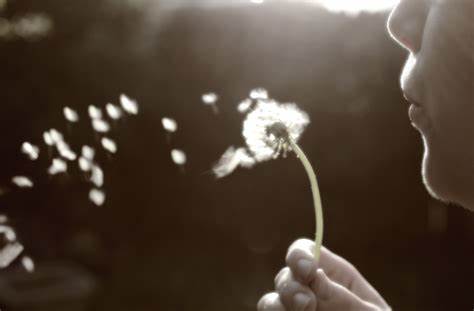

Supplement: Supplemental Information 4 [file peerj-cs-10-1866-s004.tgz › img/emociones/DESEO II.jfif]

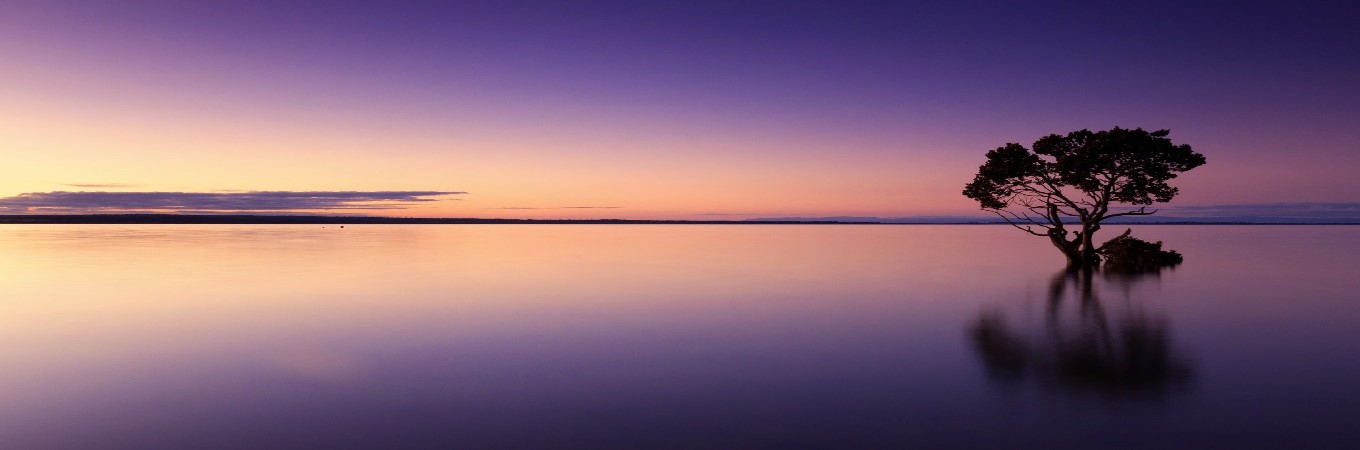

Supplement: Supplemental Information 4 [file peerj-cs-10-1866-s004.tgz › img/emociones/calma.png]

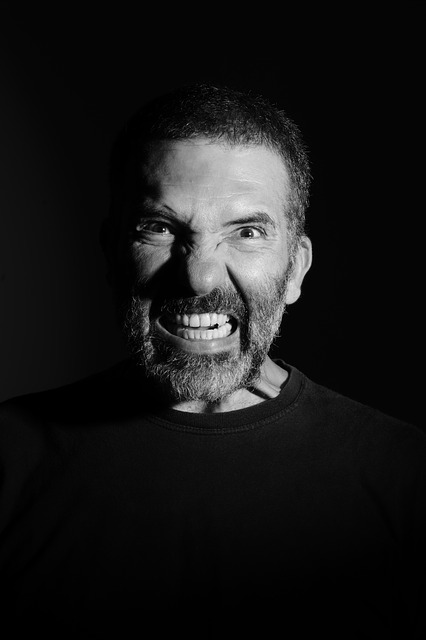

Supplement: Supplemental Information 4 [file peerj-cs-10-1866-s004.tgz › img/emociones/IRA.jpg]
